# Compendium of dyadic behavior change techniques v2.0: results from a Delphi study

**DOI:** 10.1093/abm/kaaf080

**Published:** 2025-11-07

**Authors:** Corina Berli, Karoline Villinger, Sally Di Maio, Amelie Spliesgart, Gertraud Stadler, Caterina Gawrilow, Niall Bolger, Nelli Hankonen, Aleksandra Luszczynska, Alexander J Rothman, Francine Schneider, Jeffry A Simpson, Nina Knoll, Urte Scholz

**Affiliations:** Department of Health Psychology and Behavioral Medicine, Institute of Psychology, University of Bern, 3012 Bern, Switzerland; Department of Psychology, University of Zurich, 8050 Zurich, Switzerland; Department of Psychology, Columbia University, New York, NY 10027, United States; Department of Education and Psychology, Freie Universität Berlin, 14195 Berlin, Germany; Department of Education and Psychology, Freie Universität Berlin, 14195 Berlin, Germany; Center for Prevention, Health and Human Sciences, Gender in Medicine Research Unit, Charité-Universitätsmedizin, 13353 Berlin, Germany; Department of Psychology, University of Tübingen, 72076 Tübingen, Germany; Department of Psychology, Columbia University, New York, NY 10027, United States; Faculty of Social Sciences, Tampere University, 33100 Tampere, Finland; Center for Applied Research on Health Behavior and Health (CARE-BEH), SWPS University, 53238 Wroclaw, Poland; Lyda Hill Institute for Human Resilience, University of Colorado at Colorado Springs, Colorado Springs, CO 80918, United States; Department of Psychology, University of Minnesota, Minneapolis, MN 55455, United States; Department of Health Promotion, Care and Public Health Research Institute CAPHRI, Maastricht University, 6200 MD Maastricht, Netherlands; Department of Psychology, University of Minnesota, Minneapolis, MN 55455, United States; Department of Education and Psychology, Freie Universität Berlin, 14195 Berlin, Germany; Department of Psychology, University of Zurich, 8050 Zurich, Switzerland

**Keywords:** dyadic intervention, couples, Compendium, taxonomy, dyadic behavior change techniques, intervention development, intervention reporting, mechanisms of action

## Abstract

**Background:**

Dyadic interventions involving a close other (eg, romantic partner) have gained increased awareness and shown initial promise, but a shared language and systematic approach to describing their intervention content (ie, dyadic behavior change techniques [DBCTs]) is lacking.

**Purpose:**

This study aimed to further develop a comprehensive and expert-validated Compendium of DBCTs focused on health behavior change in romantic couples to support intervention development and facilitate intervention reporting.

**Methods:**

A 2-round Delphi process with international experts (1: *N *= 20; 2: *N *= 19) was conducted. Experts rated the clarity and comprehensibility of DBCTs, as well as their expected link with the most proximal mechanisms of action. Additionally, 14 experts convened for an online discussion via video conferencing to address key issues and emerging questions.

**Results:**

The resulting Compendium of DBCTs v2.0 includes 219 DBCTs that specify who (ie, execution) does what (ie, intervention task) for whom (ie, target). DBCTs are linked to 32 hypothesized most proximal mechanisms of action. An interactive Webtool (www.dbctcompendium.com) was created to facilitate access to and use of the Compendium.

**Conclusions:**

The Compendium of DBCTs v2.0 offers a classification of DBCTs validated through expert consensus. It supports systematic development and reporting of dyadic interventions aimed at health behavior change in couples by specifying hypothesized links with underlying mechanisms of action. Future research should focus on identifying the effectiveness of DBCTs under various conditions and the Compendium’s applicability to other dyad types and behavioral domains.

## Introduction

Picture a romantic couple, Taylor and Jamie, who love to spend their time having dinner and watching a movie together. When Taylor starts going for an evening run to improve physical fitness, their partner Jamie might decide to join, cheer them on, or negotiate how it fits into their shared routine. Attempts to change one’s behavior often unfold in a social setting, shaped by our daily interactions with others. “Others” are not limited to romantic partners; they may also be siblings, friends, work colleagues, parents, roommates, or children. Recognizing the importance of these relationships, many researchers have increasingly involved a close other, such as a romantic partner, family member, or peer, in efforts to change behavior^1–3^ known as dyadic interventions. We define a dyadic intervention as an intervention that engages both members of a dyad with the aim of influencing an outcome in one or both members, with a dyad being any social group of 2 people who interact and influence each other.^4^ Dyadic interventions typically involve non-professional dyads such as romantic couples, two adult family members, parent-child dyads, peer dyads, but may be instructed by a (professional) interventionist.

One important issue in the field of research on dyadic interventions aimed at behavior change is that there is no shared language regarding how to report the content of these interventions (eg, what dyad partners are instructed to do). Also, reports of dyadic interventions often do not clarify what key theoretical determinants^5^ or mechanisms of action^6^ of health behavior change they attempt to modify by using certain techniques. To establish what makes dyadic interventions effective, we need to be able to identify dyadic behavior change techniques (DBCTs) more accurately and determine through which mechanisms of action they might drive behavior change.

As a first step forward, Di Maio et al^7^ developed a Compendium of Dyadic Intervention Techniques v1.0 for romantic couples as one of the most important relationships during adulthood. The Compendium v1.0 provides a comprehensive and systematic description of existing DBCTs based on a systematic review of dyadic interventions with romantic couples aimed to change a health behavior. In the present paper, we offer an extended and revised version of the Compendium v2.0 with the goal of facilitating the reporting and development of dyadic behavior change interventions in couples. By means of a 2-round Delphi process with international experts in dyadic behavior change, we assessed acceptance of and agreement on the Compendium.

During the final step of revising the Compendium v2.0, we decided to switch from the original term “dyadic intervention technique (DIT)” as used in the Compendium v1.0^7^ to “DBCT” to reflect the focus on techniques directed toward behavior change. Analogously, we now refer to “determinants” as used in the Compendium v1.0 as “mechanisms of action.” To avoid confusion, we consistently refer to the new terminology throughout the manuscript.

### What are the active ingredients of dyadic interventions?

Several reviews summarize the evidence of dyadic interventions in close relationships for health behaviors,^1^ focusing on physical activity, sedentary behavior,^8^^,^^9^ or HIV prevention.^10^^,^^11^ A meta-analysis by Carr et al^8^ showed small positive effects of dyadic interventions on physical activity and sedentary behavior across different dyad types. Larger effects were observed when partners shared the goal for one member, when peer/friend dyads were used, with treatment-as-usual control conditions, or in clinical samples.^8^ While these findings offer initial insights into conditions under which dyadic interventions might be effective, other sources of variation remain underexplored. One aspect that has received surprisingly little attention is how partners are involved and instructed to interact within dyadic interventions—a potential key component of their effectiveness.

The lack of systematic reporting of intervention content is a common issue in behavior change research and has been extensively discussed over the past decades.^12–14^ Behavior change techniques (BCTs) were developed to identify the active components of multicomponent interventions, primarily those targeting individuals without involving a dyad partner.^13^^,^^14^ BCTs are typically delivered by professionals or can be self-enacted.^15^ While BCTs can capture tasks in dyadic interventions where both partners are individually targeted (eg, both are instructed to self-monitor their own behaviors), they fall short in describing techniques that involve partner interactions.^7^^,^^16^

The meta-analysis by Carr et al^8^ did not rely on coding BCTs.^13^^,^^14^ However, it is questionable whether using individual BCTs would have resulted in a precise description of dyadic intervention content or a meaningful differentiation between dyadic and individual conditions. For instance, in studies comparing individual with dyadic and collaborative planning^17–20^ all conditions would have been coded with the BCT “planning,”^14^ missing critical distinctions (eg, whether one or both dyad partners plan for either one target person or for them as a dyad). Moreover, techniques specific to the dyadic context, such as mobilizing support from the other dyad partner or discussing preferred support strategies as a dyad for one partner, may not have been coded with enough accuracy or missed altogether.

Arden-Close et al^1^ reviewed couple-based interventions for chronic illness and found that only 3 of 14 studies reported specific couple-based techniques. Many failed to detail intervention content, possibly due to the lack of a standardized framework for reporting dyadic intervention content. Beyond describing the task (eg, goal setting), it is crucial to specify who is involved in the goal setting (ie, one partner or the dyad) and for whom the goal setting is being done (ie, one partner or the dyad). A shared and systematic way of precisely reporting the dyadic interaction, that is, DBCTs, is of key relevance for advancing understanding of what works in dyadic interventions. A DBCT (formerly DIT) is defined as “an observable and replicable intervention technique that explicitly involves any form of interaction with, or clear reference to, a non-professional dyad partner to change behavior” (p. 6).^7^ Although a professional (ie, interventionist) may guide the process, the technique itself requires dyad partners to interact.

### What are the mechanisms targeted by dyadic interventions?

Beyond identifying and reporting DBCTs, little is known about the underlying social or psychological mechanisms these techniques aim to influence. Identifying modifiable “putative targets” that drive the actual behavior change is a key step in the experimental medicine approach.^21^^,^^22^ These targets, or *mechanisms of action* (MoAs),^6^ can be defined as theoretical constructs that represent the processes through which a technique affects behavior, and are sometimes referred to as *determinants*, the causal antecedents of behavior.^5^ We propose that each DBCT engages a *most proximal MoA* directly triggered by the task being performed. For example, reviewing past successes may primarily activate self-efficacy. However, a DBCT may also engage *distal* MoAs, activating downstream or parallel mechanisms, depending on how the intervention task is performed in the dyadic context. For example, reviewing past successes together with the partner may, apart from self-efficacy as most proximal MoA, also set off social support processes between partners^23^ as *distal* MoAs. Distal MoAs tend to be less task-specific and may apply across multiple DBCTs.

As noted in prior reviews,^1^ dyadic interventions often lack a clear theoretical rationale. Only half of the dyadic interventions included in the systematic review by Di Maio et al^7^ explicitly reported a theoretical basis, most commonly social cognitive theories, followed by humanistic and socio-ecological approaches.^24^ Most studies introduce a dyadic or social construct (eg, social support, dyadic efficacy, relationship functioning). Overall, it remains unclear how the theories were used to specifically inform the dyadic component of the intervention. Yet theory is essential for identifying which MoAs to target, guiding intervention design, and explaining why an intervention is effective.^25^ It also provides a basis for testing and refining behavioral theories.^26^ While interventions guided by behavioral theory have shown robust effectiveness,^27^ meta-analyses and reviews suggest that merely stating a theoretical basis does not necessarily improve outcomes.^28^ This may be because “theory-inspired” interventions fail to link intervention components with theory.^28^ Evidence from physical activity interventions among breast cancer survivors, for example, shows that greater integration of theory throughout intervention development, implementation, and evaluation is associated with larger effect sizes.^29^

However, with some exceptions, theories rarely specify the techniques with which to target MoAs. Thus, articulating hypothesized links between DBCTs and most proximal and distal MoAs is essential for theory-driven intervention development.^5^ Currently, MoAs in dyadic interventions are rarely well-specified, measured, or tested. Some studies examine the effects of dyadic interventions on specific MoAs, such as planning or social control^30^ or test action control, social support, or social control as mediators.^17^^,^^18^^,^^31^ MoAs are typically defined at the intervention condition level—often involving multiple techniques—rather than at the technique level. For instance, in a dyadic intervention couples might be instructed to set physical activity goals for a target person, and subsequently, one partner sends text message reminders to the target person, with action control and social support proposed as mediators.^24^

Thus, we currently lack sound empirical evidence on the specific MoAs elicited by particular DBCTs. A first step toward advancing this understanding is to better specify hypotheses about which most proximal and distal MoAs each DBCT is expected to trigger. This will enable their measurement and testing, supporting theory development and the design of more effective dyadic interventions.

### The Compendium of Dyadic Behavior Change Techniques (DBCTs; formerly DITs)

**Figure 1.**
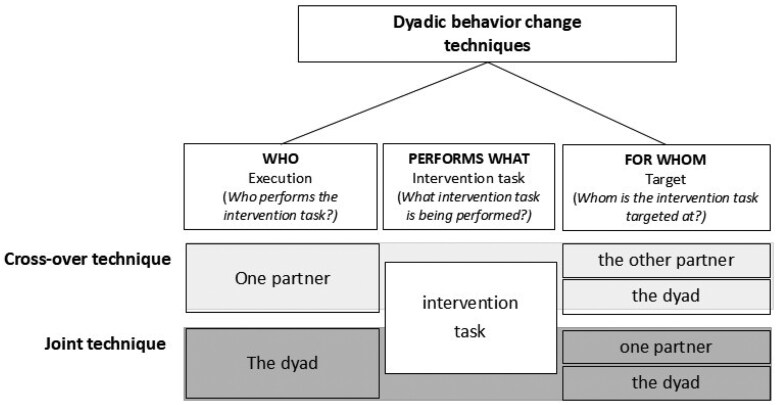
Types of dyadic behavior change techniques (DBCTs) adapted from Di Maio et al.^7^

To address these shortcomings, Di Maio et al^7^ developed a systematic framework for describing DBCTs (formerly DITs) in behavior change interventions with romantic couples. Each DBCT specifies *who* (execution level) *performs what* (intervention task) *for whom* (target level; see Figure 1).

Two main types of DBCTs can be distinguished: *cross-over techniques*, performed by one partner for the other partner or the dyad; and *joint techniques*, performed by both partners for one partner or the dyad. Combining these types with the possible targets yields 4 DBCT versions per intervention task. For instance, the task “creating a coping plan” can be applied in 4 ways—one partner creates a coping plan for the other or the dyad, or the coping plan is created jointly by both for one partner or the dyad. While joint techniques require active collaboration, cross-over techniques may involve interaction (eg, monitoring or support) or a mental representation of the other partner (eg, considering a partner’s perspective). Cross-over techniques can also be reciprocal, with both partners assigned complementary roles—termed *mutual* cross-over techniques. For more examples of DBCTs, see the electronic supplementary materials.

Drawing on a systematic review of health behavior change interventions with romantic couples,^7^ a first version of the Compendium of DBCTs v1.0 was developed, describing 160 DBCTs across 76 unique tasks. About two-thirds of these techniques were distinct from existing BCTs as defined in existing taxonomies.^5^^,^^14^ DBCTs were linked to 30 hypothesized most proximal MoAs (in v1.0 referred to as “determinants” of behavior change), nested in 10 domains of the theoretical domains framework (TDF).^32^^,^^33^ The TDF is an integrated theoretical framework that clusters cognitive, affective, social, and environmental influences on behavior clustered into overarching domains.

While not exhaustive or limited to effective techniques, the Compendium provides a structured approach to reporting DBCTs and supports the development of dyadic interventions by linking techniques to MoAs. However, further refinement is needed. This includes: (1) adding theory-driven DBCTs currently missing from the Compendium, (2) integrating DBCTs and MoAs within socio-ecological or dyadic theories, (3) improving usability by providing clearer examples, and (4) reaching broader expert consensus, as the initial development was led by researchers in Switzerland and Germany.

### Aims of the study

To enhance acceptance and use of the Compendium within the scientific community, this study aimed to establish expert consensus on whether: (a) the listed DBCTs accurately reflect DBCTs; (b) they are clear and understandable; (c) they are hypothesized to stimulate the proposed most proximal MoAs; and (d) the Compendium is useful for intervention development and reporting. Using a Delphi study, we sought to refine Compendium v1.0^7^ into a more widely accepted and agreed-upon Compendium of DBCTs v2.0 that facilitates the development and reporting of behavior change interventions in couples.

## Methods

We used a Delphi study, a formal consensus method,^34^ to examine agreement on the identified DBCTs and overall utility of the Compendium. Following procedures used in previous taxonomy development,^14^ the Delphi study consisted of 2 feedback rounds using online questionnaires and a subsequent online discussion via videoconferencing. The online discussion served to clarify and discuss remaining questions and emerging issues. The study adhered to the principles outlined in the Declaration of Helsinki^35^ and received ethical approval from the ethics committee of the University of Zurich in February 2020 (Nr. 20.2.2). Prior to participation, all participants gave written informed consent.

### Participants

**Figure 2.**
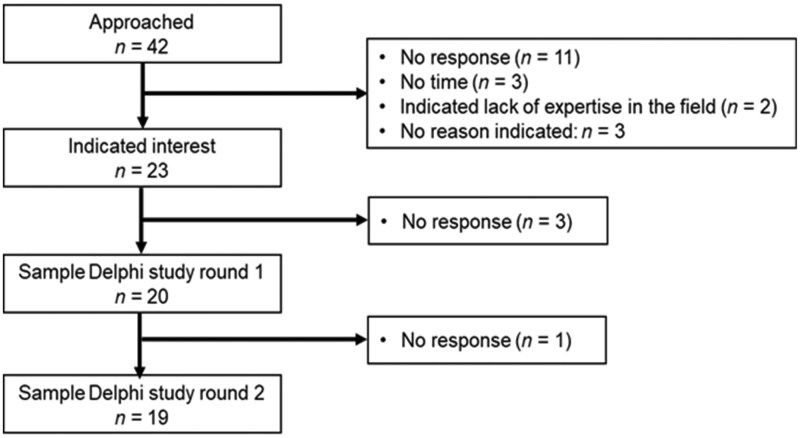
Participant flow.

We recruited international scholars with expertise in developing, implementing, and evaluating dyadic health behavior change interventions. Experts were identified through various scientific networks, and members of the project’s Advisory Board were also invited. Out of 42 experts contacted via email, 23 expressed interest (55% response rate), and 20 participated in the first round. The three who did not participate did not provide reasons (see Figure 2).

Among the 20 participants, 85% were women, with an average age of 50 years (SD = 11.65). Thirteen were based in North America (65%) and 7 in Europe (35%). Nineteen held senior academic positions (13 full professors, 4 associate professors, and 2 assistant professors), and one was a postdoctoral researcher. All had experience in designing or conceptualizing dyadic health behavior change interventions; 80% had implemented, and 75% had evaluated such interventions.

Expertise spanned various health behaviors, with the most common being physical activity (*n* = 12) and healthy eating (*n* = 10), followed by medication adherence (*n* = 4), sexual/protective risk behavior (*n* = 3), smoking (*n* = 2), alcohol/substance use (*n* = 2), and other areas like chronic illness management and weight control (*n* = 7). All 20 experts were invited to the second round, with 19 participating (95%). Each participant received CHF 100 (USD 107) per completed round. Additionally, 14 experts (70%) joined an online discussion held within 2 months after the second round.

### Procedure

The introductory materials (booklets) that were developed for the Delphi process and the evolving Compendium versions v1.1, v1.2, and v2.0 with highlighted changes can be found on Open Science Framework (https://osf.io/r43v6/).

#### Step 1: expanding the Compendium of DBCTs v1.0

To enhance theoretical integration and usability, we expanded the Compendium of DBCTs (formerly DITs) v1.0. First, we identified socio-ecological theories, which account for the interplay between individual, relational, and contextual factors, and dyadic theories, which explicitly model interdependence between partners through team and advisory board input, as well as from studies in the original systematic review.^7^ We did not restrict this collection to formal theories, but also included theoretical frameworks, concepts, or evidence-based behavior change approaches, if relevant. Individual behavior change theories without a social component were excluded, as they are well-documented elsewhere. These theories were reviewed for additional DBCTs (top-down approach) and mapped to relevant most proximal MoAs or specific DBCTs by S.D. and K.V. and then reviewed by the project team (C.B., G.S., C.G., N.K., U.S.). Consensus was achieved through team discussions. Second, we developed illustrative examples for each DBCT to improve clarity. Third, DBCTs on support regulation (eg, plan to provide support to, practice support behavior for, set goals to provide support to, etc) were reorganized as a separate table of DBCTs aimed to change *support behavior* for health behavior change, nested within the TDF domains. For example, practicing support behavior was linked to the MoA behavioral skills within the domain skills. Lastly, minor wording adjustments were made to intervention tasks, MoAs, and domain classifications. These refinements resulted in the updated Compendium v1.1.

#### Step 2: first round of the Delphi study

Between June and August 2023, experts received personalized links along with a pseudonymized ID to complete an online survey (approx. 90–120 minutes). After reviewing study details and providing informed consent, participants had 6 weeks to respond, with up to 3 reminders sent.

The survey collected demographic and professional background information, followed by an introduction to DBCTs and the Compendium v1.1. Experts were able to download the introduction material (booklet for round 1) and Compendium v1.1. Each expert evaluated 14 to 16 sets of DBCTs (from 74 total), ensuring each was reviewed by at least 3 experts. For each of the DBCTs within a set, experts were asked to indicate whether they (a) represented a DBCT and (b) were clear and comprehensible. Furthermore, they were asked to rate whether (c) the presented set of DBCTs is expected to stimulate the proposed most proximal MoA. Note that during both rounds of the Delphi Study, we used the terms “dyadic intervention technique (DIT)” and “determinants” to refer to DBCTs and MoAs, respectively. Experts were invited to share any suggestions or concerns about the DBCTs in an open-text format. Subsequently, they were asked to rate the overall utility of the Compendium for intervention development and reporting and the likelihood of using it in their own work. All ratings were assessed using a 4-point Likert scale from “strongly disagree” (1), “disagree” (2), “agree” (3) to “strongly agree” (4) and if experts did not strongly agree, they were asked to provide explanations. Finally, experts were prompted to suggest any additional DBCTs not yet included in the Compendium or suggest any necessary changes. They were asked to assess the difficulty of rating the DBCTs on a 4-point Likert scale ranging from “not difficult at all” (1) to “very difficult” (4). Feedback from this round informed revisions, leading to Compendium v1.2.

#### Step 3: second round of the Delphi study

Conducted between January and March 2024, the second round invited the previous participants to complete a 60- to 90-minute online survey within 5 weeks, with up to 3 reminders. Experts received a summary of revisions made to the Compendium v1.2, with changes highlighted and detailed explanations provided (booklet for round 2). They were asked to re-evaluate 60 DBCTs and 21 most proximal MoAs that had received ratings below 3 from at least one expert (indicating “disagreement” or “strong disagreement”), alongside newly added techniques (*n *= 3). Each expert only assessed the sets of techniques they had assessed in the first round, averaging 11 DBCTs (range: 4-18) and 2 MoAs (range: 0-4), using the same procedure as in the first round. Additionally, participants rated the Compendium’s overall utility and difficulty of rating. They were prompted to suggest potential distal MoAs, new DBCTs, or relevant theories not yet included.

#### Step 4: expert discussion

We held 2 content-parallel online discussion rounds in April and May 2024 with 6 and 8 experts, respectively. Each session followed the same structure, was recorded via Zoom, and was transcribed for further analysis. In the first part, experts were briefed on revisions made after Round 2 of the Delphi study and informed about upcoming steps. They could raise questions and clarify any points. In the second part, we invited experts to discuss key issues from the Delphi feedback, which were guided by structured questions and moderated by the project team. Discussion questions focused on how to improve the Compendium’s usability for intervention reporting and development and enhance user-friendliness and to identify key moderators of effectiveness.

#### Step 5: finalizing the Compendium of DBCTs v2.0 and Webtool

Incorporating feedback from Round 2 and the expert discussions, we finalized the Compendium v2.0. We also launched a website introducing the Compendium and its development, which includes an interactive Webtool to explore and filter its contents (www.dbctcompendium.com).

## Results

Table 1 summarizes the composition of the different versions of the Compendium throughout the development process. For detailed changes to the Compendium in v1.1, v1.2, and v2.0, see Open Science Framework (https://osf.io/r43v6/). The Compendium v2.0 of DBCTs can be found in the electronic supplementary materials.

**Table 1.** Composition of the Compendium of DBCTs throughout the development process.

Compendium version	v1.0	v1.1	v1.2	v2.0
DBCTs	160	260	230 (HB-180 + S-50)	219
Intervention tasks	76	74	76 (HB-63 + S-13)	73
MoAs	30	38	39 (HB-29 + S-10)	32
Domains	10	15	15 (HB-10 + S-5)	10

Abbreviations: DBCTs, dyadic behavior change techniques; MoAs, mechanisms of action; HB, health behaviors.

Compendium v1.2 contained a main table with DBCTs related to health behaviors (denoted as HB) and a separate table with DBCTs related to support behavior for behavior change (denoted as S). In v1.0, v1.1, and v1.2, DBCTs and MoAs were originally referred to as DIT and determinant, respectively. For detailed changes to the Compendium in v1.1, v1.2, and v2.0, see Open Science Framework (https://osf.io/r43v6/).

### Step 1: expanding the Compendium of DBCTs v1.0

The resulting Compendium of DBCTs v1.1 described 260 DBCTs with 74 unique intervention tasks, hypothesized to be linked to 29 MoAs that were in turn nested in 10 domains of the TDF.^32^^,^^33^ Overall, 34 different socio-ecological or dyadic theories were listed and linked to the DBCTs.

### Step 2: first round of the Delphi study

**Figure 3.**
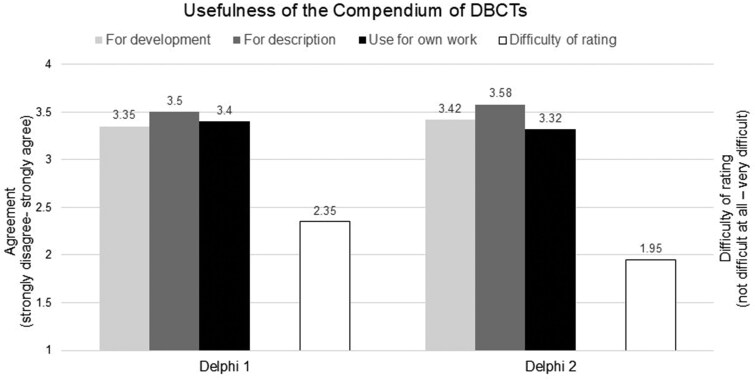
Rating of the usefulness of the Compendium of dyadic behavior change techniques (DBCTs) and perceived difficulty of rating during Delphi study round 1 (left) and Delphi study round 2 (right).

Results of the first round of the Delphi study indicated that the rating of the utility for development (*M *= 3.35, SD = 0.65) and description (*M *= 3.53, SD = 0.50) of dyadic interventions as well as the likelihood to use the Compendium in one’s own work (*M *= 3.4, SD = 0.66) was overall positive (see Figure 3). Out of 20 experts, 18 (90%), 20 (100%), and 18 (90%) indicated that they agreed or strongly agreed that the Compendium is useful for intervention development, description, and for their own work, respectively. The difficulty of rating was below the scale mean (*M *= 2.35, SD = 0.85), with lower values indicating lower difficulty. Eight experts (40%) found it difficult or very difficult to rate DBCTs.

Out of the 260 DBCTs that were evaluated by the experts, 19 received an average rating below 3 (range: 1.50-2.75), indicating that some experts either “disagreed” or “strongly disagreed” to the statement whether it is considered a DBCT, and 24 received an average rating below 3 (range: 1.75-2.80) regarding the question whether a respective DBCT is clear and comprehensible. Additionally, 9 out of 38 MoAs received a rating below 3 (range: 2.00-2.80) for the question whether the presented set of DBCTs stimulates the proposed MoA for behavior change. All DBCTs and MoAs with low agreement were carefully examined and revised.

Based on the experts’ comments, comprehensive changes were made regarding the labels and clustering of the intervention tasks, MoAs, and phrasing of the examples. Table 2 summarizes the amendments made. Two separate compendium tables were created: a main table for DBCTs focused on health behaviors (eg, one partner plans [a health behavior] for the other partner) and a separate table for DBCTs focused on support behavior to help with health behavior change (eg, one partner plans to provide support to the other partner) to underscore their distinct focus. Additional theories that were mentioned as relevant for DBCTs by experts were incorporated into the Compendium. Some of the specific comments made by the experts highlighted the undefined role of the interventionist in examples presented in the Compendium v1.1.

**Table 2.** Summary of changes made to the Compendium of DBCTs after the first and second rounds of the Delphi study.

	Delphi study round 1	Delphi study round 2
Comments received (total)	603	42
Changes made (total)	357	126
Intervention tasks added	3	1
Intervention tasks revised	20 (+9 language revision)	16
Intervention tasks combined	2	2
Intervention tasks removed	0	2
Intervention tasks transferred to different domain	5	14
Examples revised	237	120
MoAs added	1	1
MoAs revised	5	15
Domains added	1	0
Domains removed	1	0
Theories added	31	3

Abbreviations: DBCTs, dyadic behavior change techniques; MoAs, mechanisms of action.

The examples for each DBCT were phrased in an active present tense to precisely define the roles of each partner. In line with suggestions from the experts, we revised each example to include the instructional nature of the DBCTs, clarifying the involvement of an interventionist in that all DBCTs are guided or instructed as part of an intervention. We created a booklet summarizing and addressing important issues raised in round 1 (booklet round 2), providing, for example, conceptual clarifications regarding DBCTs and MoAs and constraints of the Compendium (eg, effectiveness of DBCTs).

The resulting revised Compendium of DBCTs v1.2 included a main table with DBCTs focused on changing health behaviors that described 180 DBCTs with 63 unique intervention tasks, hypothesized to be linked to 29 most proximal MoAs that were nested in 10 domains of the TDF. A separate table with DBCTs focused on changing support behavior included 50 DBCTs with 13 unique intervention tasks, hypothesized to be linked to 10 MoAs that were in turn nested in 5 domains. Overall, 39 different socio-ecological or dyadic theories were assigned to the DBCTs and MoAs in the Compendium.

### Step 3: second round of the Delphi study

The Compendium of DBCTs v1.2 was rated positively for its utility in both the development (*M *= 3.42, SD = 0.59) and description (*M *= 3.58, SD = 0.58) of dyadic interventions, as well as its perceived potential usefulness for the experts’ own work (*M *= 3.32, SD = 0.57; see Figure 3). Out of 19 experts, 18 (94.7%) indicated that they agreed or strongly agreed that the Compendium is useful for intervention development, description, and for their own work. The perceived difficulty of rating improved slightly to *M *= 1.95 (SD = 0.76) following revisions made after the initial round of the Delphi study. Out of 19 experts, 3 (15.8%) found it difficult or very difficult to rate DBCTs.

Out of 106 DBCTs that were re-evaluated regarding the question of whether it is considered a DBCT, 24 received an average rating below 3, indicating that experts either “disagreed” or “strongly disagreed.” Out of 134 DBCTs that were re-evaluated regarding the question of whether it is clear and comprehensible, 13 DBCTs received an average rating below 3. Furthermore, 4 out of 9 MoAs that were re-evaluated for the question of whether the presented set of DBCTs stimulates the proposed MoA for behavior change received an average rating below 3. Any DBCT and MoA of the Compendium v1.2 that did not yield agreement among the experts (ie, rating below 3) were revised accordingly based on discussion within the project team.

Beyond specific feedback regarding DBCTs, several overarching themes emerged. One was the question of whether the dyad could provide support to itself. To clarify, we redefined support-related techniques as cross-over (one partner supporting the other), while still allowing for mutual support (ie, one partner can support the other and vice versa). In illustrative examples, we also replaced generic references to people with names (ie, Alex and Billie) to improve clarity. Experts found the separate tables for DBCTs focused on health behavior (eg, set a goal for) and support behavior (eg, set goal to provide support to) confusing, so we merged them, classifying support-related techniques under “support provision skills” MoA. Overlapping tasks (eg, set goal for health behavior and set goal to provide support to) are now cross-referenced. Additionally, experts raised concerns about the effectiveness of techniques and potential moderators of effectiveness. Feedback also suggested a field of tension: some favored the Compendium’s detailed approach; others suggested streamlining for usability. These issues were further explored during the expert discussions.

Finally, experts suggested potential distal MoAs that DBCTs might influence, including social exchange processes (eg, informational and emotional support, support quality via communication about goals), individual and dyadic regulation (eg, self-efficacy due to modeling, motivation, feelings of (couple) autonomy, but also shared goals, shared norms or shared appraisals as well as collaborative problem-solving), and relationship processes (eg, accountability, commitment, intimacy, relationship satisfaction, reciprocity, empathy, and perspective-taking). A complete overview of the distal MoAs can be found in the supplemental material (ESM 2).

### Step 4: expert online discussion

Several important suggestions emerged from the 2 content-parallel online discussions attended by a total of 14 out of the 20 experts. Experts commented on the importance of clarifying that the listed techniques in the Compendium do not guarantee effectiveness and of clearly presenting the limitations of the Compendium. Experts also pointed out the need for considering contextual factors, collectively suggesting diverse factors they consider as important moderators of the effectiveness of dyadic interventions in general and DBCTs in particular. Furthermore, the importance of increasing visibility and accessibility was stressed by experts, particularly for researchers unfamiliar with dyadic interventions. It was recommended by experts to include prototypical examples of dyadic behavior change interventions that illustrate how to report DBCTs. It was further recommended to provide links to studies that made use of respective DBCTs. Experts agreed that a webpage containing such information is helpful.

Moreover, opportunities of the Compendium for future research and practice were discussed that could be pursued as a future avenue. Several experts suggested that decision trees or flowcharts could be useful to assist researchers in selecting different features of DBCTs (eg, execution or target level, type of intervention task). Additionally, experts suggested the benefit of indicating which DBCTs could be used together (eg, goal setting and planning) to enhance effectiveness and differentiating between basic DBCTs (ie, suitable for any dyad) as opposed to more advanced DBCTs (ie, requiring certain skills and prerequisites).

### Step 5: finalizing the Compendium of DBCTs v2.0 and Webtool

After validating the Compendium through expert consensus, the final revised Compendium v2.0 (see electronic supplementary materials) consisted of one table describing 219 DBCTs with 73 unique intervention tasks, hypothesized to be linked to 32 most proximal MoAs that are nested in 10 domains of the TDF.^32^^,^^33^ Overall, 37 different socio-ecological or dyadic theories were connected to the different DBCTs and MoAs in the Compendium v2.0. Please note that during this final step of revising the Compendium, we decided to adapt the original terminology and to use the terms “dyadic behavior change technique (DBCT; formerly DITs)” and “mechanisms of actions (MoAs; formerly determinants)” from v2.0 on. This reflects more closely the focus of the Compendium on techniques aimed at behavior change.

To address the point raised by experts that the Compendium’s limitations need to be transparent, we provided an overview of what the Compendium can and cannot do along with the Compendium v2.0. To improve the comprehensibility and user-friendliness of the Compendium of DBCTs as suggested by experts, we developed a website (www.dbctcompendium.com) to introduce the Compendium of DBCTs to a broader audience. The website provides the latest version of the Compendium of DBCTs v2.0 to be downloaded as a PDF and features a Webtool that allows to search and filter for specific DBCTs according to one’s needs. The interactive Webtool is designed to simplify the use of the Compendium and ensure its accessibility. The website further illustrates examples of how to utilize the Compendium for both developing and reporting dyadic interventions as a response to the suggestions from experts during the online discussion.

## Discussion

To address the lack of a shared language for reporting the content of dyadic intervention and to facilitate the development and reporting of dyadic interventions, we further developed an agreed-upon Compendium of Dyadic Behavior Change Techniques v2.0 for health behavior change in couples. During a 2-round Delphi process and subsequent online discussion with experts in planning, implementing, and evaluating dyadic interventions, DBCTs were added, removed, combined, or revised.

The Compendium of DBCTs v2.0 includes 73 intervention tasks in 219 versions of DBCTs specifying *who performs what for whom*. The DBCTs are hypothesized to be linked to 32 most proximal MoAs, which are nested in 10 domains of the TDF,^32^^,^^33^ covering a broad range of behavior change processes, from knowledge and beliefs about capabilities to goal setting, behavioral regulation, and social influences. Experts rated the Compendium v2.0 overall as useful for intervention description and development and for their own work. To support the dissemination and accessibility of the Compendium and its use for research, we set up a website (www.dbctcompendium.com) including a Webtool to navigate through the extensive collection of DBCTs.

Existing frameworks such as the original BCT taxonomy,^14^ the behavior change techniques ontology (BCTO^13^), the mode of delivery ontology (MoD^36^), and the compendium of self-enactable techniques^15^ specify various BCTs and delivery methods (eg, face-to-face) but are not focused on the ways 2 persons (ie, a dyad) are instructed to engage in an intervention task. Similarly, while the Intervention Mapping taxonomy^5^ acknowledges the role of social agents and important social influences, it does not provide a structured approach to classifying BCTs involving 2 people. The Compendium of DBCTs v2.0 serves as an additional resource complementing existing classification systems to specify BCTs, yet with a specific focus on interventions in dyads such as romantic couples.

The revised Compendium v2.0 builds on its earlier version v1.0,^7^ which was based on a systematic review of dyadic health behavior change interventions with romantic couples. The Compendium of DBCTs v2.0 includes several key updates: (1) DBCTs were added using a “top-down” approach based on literature; (2) socio-ecological and dyadic theories associated with DBCTs and MoAs were added to facilitate development of interventions; (3) DBCTs were numbered to facilitate reporting; (4) DBCTs were complemented with illustrative examples to enhance comprehensibility and distinctiveness. These examples particularly help to clarify distinctions between different DBCTs of the same intervention task. For example, monitoring the other dyad partner’s health behavior (DBCT #40a: Billie is directed to monitor and record Alex’s blood pressure) is distinct from monitoring the dyad’s health behavior (DBCT #40b: Billie is asked to keep track of their consistent use of condoms); (5) another main change is that DBCTs regarding support interactions were specified as cross-over techniques (ie, one partner provides support to the other partner). Exceptions apply when the dyad can jointly execute a support task for one dyad partner, eg, practicing skills to provide support to one dyad partner (DBCT #60) or strengthening confidence to provide support to one dyad partner (DBCT #62). As mentioned in the introduction, partners being instructed to provide support in a reciprocal way would be captured by specifying each dyad partner separately as a target of a cross-over technique (ie, mutual cross-over techniques).

The Compendium of DBCTs v2.0 presents the first approach to systematically and precisely characterize the potential active ingredients of dyadic interventions by specifying existing DBCTs aimed at health behavior change in romantic couples. Importantly, the Compendium does not identify DBCTs that have empirically proved to be effective, but rather describes DBCTs existing in theory and previous intervention research as a first step toward establishing effectiveness. How you navigate through the Compendium v2.0 depends on what you want to use it for. When *developing* dyadic intervention content, researchers can start by identifying important socio-ecological or dyadic theories, domains, or MoAs through comprehensive literature search (eg, as part of existing intervention development frameworks^37^). The Compendium v2.0 then presents hypotheses regarding the links between DBCTs and the selected MoAs, domain, or theory. Once the DBCTs have been selected, researchers next need to make decisions about what versions of DBCTs to use (eg, who performs the intervention task? Who is the target of the intervention task?). Ideally, this should again be guided by theory or specific theoretical assumptions and described in intervention reports. When *reporting* dyadic intervention content, researchers can use the Compendium to precisely report all DBCTs used by describing who performs the intervention task (ie, one partner or the dyad), what intervention task is performed, and for whom the intervention task is being performed (ie, for one partner or the dyad), using the number codes in the Compendium. Second, they should indicate the most proximal (and, if relevant distal) MoAs that each of the DBCTs targeted to change behavior. Third, they should outline whether and which theories were at the basis for choosing the specific DBCTs. See examples of how to use the Compendium on the webpage (www.dbctcompendium.com). This approach to precise reporting aligns with broader efforts to improve the transparency and reproducibility of behavioral interventions and can be guided by existing frameworks such as the Template for Intervention Description and Replication (TIDieR) checklist^38^ or the Theory Coding Scheme.^39^

We also expect the Compendium of DBCTs v2.0 to evolve further. The initial Compendium v1.0 was developed based on a systematic review of the literature of interventions with romantic dyads, but its applicability is not necessarily restricted to romantic dyads, and we expect that it can be extended or adapted to encompass DBCTs relevant for other dyad types involving close others, such as friend or peer dyads,^40^^,^^41^ family dyads,^20^ or parent-child dyads.^42^ The applicability to other dyad constellations is also relevant for intervention studies that might not focus on a specific dyad type, but allow for variations in dyad constellations^20^ and for summarizing evidence across different dyad types.^8^ Depending on the dyad types, some DBCTs might be more and others less frequent or relevant. For example, in interventions with parent-child dyads aimed to change a child’s health behavior, the use of cross-over and joint DBCTs that target the child might vary in effectiveness.^19^ As an important next step, the applicability of the Compendium for coding intervention content of interventions with other types of dyads needs to be established. Similarly, identifying DBCTs might also be relevant in other health or non-health contexts (eg, coping, academic behavior change, problematic behavior change). For instance, reviews on dyadic interventions in the context of coping with cancer^43^^,^^44^ highlight similar challenges found in health behavior change interventions, namely, the lack of clear criteria for summarizing intervention content. While the format of an intervention (eg, group-based) is also important, identifying *what* is done within the intervention is crucial to understand its success.

### Limitations

The Compendium of DBCTs v2.0 has several limitations. First, the inclusion of a DBCT does not indicate that it was proven to be an effective DBCT. This remains an empirical question requiring future testing, beyond theoretical evidence for their relevance. However, a systematic and shared description is a necessary first step toward establishing effectiveness of specific DBCTs or sets of DBCTs. Second, the Compendium v2.0 is not comprehensive in terms of listed DBCTs, MoAs, or associated theories. Each DBCT may elicit multiple or distal MoAs (eg, social support or control, relationship well-being, accountability or shared appraisals), but repeating those for each DBCT was deemed inefficient. Future work should clarify which types of distal MoAs are likely to be elicited by specific DBCTs, similar to the approach taken by Michie et al.^45^ Future work could also attempt to categorize theories further into classes of theories relevant for different MoAs. Third, complete distinctiveness of DBCTs cannot be guaranteed. To balance clarity and usability, we integrated elements from both development and coding taxonomies. As emphasized by Kok et al,^5^ careful matching of techniques with mechanisms remains essential. Fourth, the Compendium’s length and complexity may challenge users. We opted for higher-level resolution to avoid losing meaningful nuances. With more empirical data, future versions can refine or simplify the structure.

Methodologically, while expert participation in both Delphi rounds (*n* = 20 and *n* = 19) was adequate^14^ most experts came from Western countries (Europe and North America). This limits generalizability and reflects broader issues in psychological science shaped by Western, Educated, Industrialized, Rich, and Democratic (WEIRD) populations.^46^ The limited representation of experts from diverse cultural and socio-economic backgrounds, as well as the literature that was the basis for generating the DBCTs, may have influenced the types of DBCTs and associated MoAs represented in the Compendium and the applicability of findings beyond Western contexts.

Moreover, some decisions had to be carefully made within the project team due to the absence of standards for such methods.^14^ For example, we decided to let experts re-evaluate all DBCTs that received an average rating of below 3, indicating that experts did not or did not at all agree with the statements (1 = strongly disagree, 2 = disagree, 3 = agree, 4 = strongly agree). Despite extensive materials, some experts found DBCT rating challenging, suggesting that applying the Compendium without support could be difficult. To aid usability, the accompanying website (www.dbctcompendium.com) provides detailed guidance and examples. As uptake increases, we aim to highlight additional good-practice applications.

### Future steps

We consider the Compendium of DBCTs v2.0 as work in progress. To establish the reliability in reporting DBCTs from intervention descriptions, current efforts focus on coding dyadic interventions using the Compendium of DBCTs v2.0 with trained coders (https://doi.org/10.23668/psycharchives.16143). To refine this process, we will then develop and incorporate clear, standardized definitions for each DBCT in the next Compendium version. Additionally, to support long-term knowledge synthesis, a key future step is to represent DBCTs in a machine-readable format within an ontology, such as the Behavior Change Intervention Ontology (BCIO^47^). As part of an ongoing collaboration, we are working to create ontological definitions for DBCTs, ensuring their seamless integration into digital knowledge frameworks (https://osf.io/2364r/).

Another important next step is to establish a robust evidence base for the DBCTs, or types of DBCTs, listed in the Compendium through rigorous experimental research. As an initial effort, we are conducting a meta-analysis of empirical studies from the systematic review^7^ and beyond and 2 experimental vignette studies to assess the effectiveness of different motivational DBCTs (https://doi.org/10.17605/OSF.IO/PA7YQ). Key distinctions in the Compendium are the level of execution (ie, whether one or both dyad partners carry out an intervention task together) and target (ie, whether an intervention task is targeted at one or both dyad partners). Do these distinctions matter for effectiveness of the techniques? Early findings suggest they might. For example, planning together for one dyad partner was more effective than planning together for the dyad in increasing physical activity^20^ but less effective in lowering sedentary behavior.^48^ This, however, needs further testing across different DBCTs using experimental designs such as micro-randomized controlled trials.^49^ Additionally, exploratory expert insights from the Delphi study on distal MoAs suggest that DBCTs may influence behavior change in a unique way compared to individual BCTs, by enhancing relationship processes^17^ and fostering shared representations of goals, shared appraisals, or collaborative problem-solving. Similarly, the Dyadic Health Influence Model^50^ emphasizes an interplay between health behavior change pathways in dyads and relationship beliefs and behaviors. Moreover, evidence shows that already simple movement exercises, when performed in synchrony as a pair, resulted in more efficient collaborative problem-solving.^51^ Such proposed mechanisms, however, also require to be measured and tested to determine their role in shaping dyadic behavior change. As evidence on DBCT effectiveness grows, it can serve as a foundation for providing evidence-based recommendations on which DBCTs to use for specific purposes, a future direction identified during the expert online discussion. When assessing the suitability of DBCTs for behavior change, it is important to consider factors beyond effectiveness. These include affordability, practicability, effectiveness, acceptability, side effects/safety, and equity, collectively referred to as the APEASE criteria,^52^ which provide a structured framework for selecting and implementing interventions in real-world contexts.

As experts suggested during the Delphi process, not all DBCTs are expected to work for everyone all the time. Thus, a key question is what are moderators of effectiveness? In the intervention mapping approach, the importance of specifying the parameters of effectiveness for BCTs (“methods”) is highlighted.^5^ Based on the discussion with experts during the Delphi process, moderators of effectiveness were identified at different levels: (i) moderators at the level of the *relationship* including length of relationship, relationship quality, or shared values; (ii) moderators at the level of the *individuals* in the dyad, including diversity characteristics (eg, age, socio-economic status, gender), individual perceptions such as the investment in partner’s health^53^; and (iii) moderators at the level of the *intervention*, including for example the behavior and communication during the execution of the intervention task (eg, tone and style of how dyad partners collaborate), the health behavior context (eg, disease context or prevention), type of health behavior (eg, one-time vs daily behavior), the dyad type (eg, patient-partner) or also characteristics of the interventionist. For example, a recent meta-analysis^54^ showed that a patient-caregiver intervention was more successful in improving psychological distress if the interventionist was a psychologist compared to a nurse or a therapist. Evidence also exists demonstrating that relationship quality,^17^ types of dyad and types of goals^8^ matter for the effectiveness of dyadic interventions. Future effort should lie in identifying and testing potential moderators specific to the dyadic context, beyond frequency of intervention sessions, delivery mode, etc.^8^ Such analyses are, however, complicated by a lack of power for testing moderation given that dyadic intervention studies often have small sizes.^55^

## Conclusions

As healthcare systems face growing demands, leveraging the support of close others in health promotion presents a valuable opportunity. Yet, more work is needed to better understand what makes dyadic interventions effective. Designed to support the development and reporting of dyadic interventions, the Compendium of Dyadic Behavior Change Techniques v2.0 provides a validated, systematic classification of DBCTs for health behavior change in couples, promoting standardized reporting by specifying “who performs what for whom” and linking DBCTs to hypothesized most proximal MoAs. This supports the selection of DBCTs and identification of MoAs to measure and test. A webpage (www.dbctcompendium.com) facilitates its use. Future research should focus on evaluating the effectiveness of DBCTs, their MoAs, and the conditions under which they work best.

## Supplementary Material

kaaf080_Supplementary_Data

## Transparency statement

This study was registered as part of the overarching research project on the Open Science Framework before the project started in 2020 (https://osf.io/r43v6/). Due to the descriptive nature of the study, no analysis plan was formally pre-registered, and there is no analytic code associated with this study. De-identified data from this study and materials (surveys, introductory materials and compendium versions with highlighted changes) used to conduct the study are available in a public archive (CC BY license): https://osf.io/r43v6/.
