# Supplementary material for: Compendium of dyadic behavior change techniques v2.0: results from a Delphi study
Source: Ann Behav Med. 2025 Nov 7;59(1):kaaf080. doi: 10.1093/abm/kaaf080 (PMC12597882; doi:10.1093/abm/kaaf080)
Supplement: kaaf080_Supplementary_Data [file kaaf080_supplementary_data.zip › ESM1 CompendiumDBCTs v2.0[AU].docx]

**Compendium of Dyadic Behavior Change Techniques (DBCTs) v2.0**

Please note that in v1.0 of the Compendium, we used the terms *Dyadic Intervention Techniques (DITs)* and *determinants* to refer to *Dyadic Behavior Change Techniques (DBCTs)* and *mechanisms of action*, respectively. The current terminology was adopted during the final step of revising the Compendium v2.0, to reflect the focus of the Compendium on techniques directed towards behavior change.

| **The compendium can do…** | **The compendium cannot do (yet)…** |
| --- | --- |
| - …offer a shared and structured description of dyadic behavior change techniques by specifying “who performs what from whom” during intervention delivery and subsequent implementation. - …enhance systematic reporting of dyadic intervention content by providing structured reporting guidelines. - …propose hypothesized theoretical links of dyadic behavior change techniques with the most proximal mechanisms of action that a specific intervention task is assumed to stimulate as well as associated socio-ecological or dyadic theories. - …facilitate the development of theory-based dyadic interventions by proposing potential dyadic behavior change techniques assumed to elicit behavior change via a specific proximal mechanism of action. | - …claim effectiveness of DBCTs. - …ensure complete distinctiveness of intervention tasks, as there may be overlap. - …provide an exhaustive list of dyadic behavior change techniques: The dyadic behavior change techniques included in the present compendium are based on the results of a systematic review of the literature [BLINDED FOR PEER REVIEW] and feedback of international experts in dyadic health behavior change. However, there may be additional dyadic behavior change techniques not yet covered. - …provide an exhaustive list of associated socio-ecological or dyadic theories - …claim to be a final product, but ongoing work in progress. |

**Reference:**

This document is the electronic supplementary material of a publication accepted in Annals of Behavioral Medicine. If you wish to cite it, please cite the original publication (please note that the final citation may differ, but the DOI will remain the same):

Berli, C., Villinger, K., Di Maio, S., Spliesgart, A., Stadler, G., Gawrilow, C., Bolger, N., Hankonen, N., Luszczynska, A., Rothman, A. J., Schneider, F., Simpson, J. A., Knoll, N.*, & Scholz, U.* (2025). Compendium of Dyadic Behavior Change Techniques v2.0: Results from a Delphi Study. *Annals of Behavioral Medicine.* DOI: 10.1093/abm/kaaf080

*Shared last authorship

**Dyadic Behavior Change Techniques**

**Knowledge/Information**

Information processing (DBCT#1)

Sharing information (DBCT#2)

**Skills**

Behavioral skills (DBCT#3)

Communication skills (DBCT#4)

**Social Role and Identity**

Perspective taking (DBCT#5)

Communal orientation (DBCT#6)

Communal action (DBCT#7)

Norms (DBCT#8)

Roles (DBCT#9)

**Beliefs about Capabilities**

Self-efficacy/Dyadic efficacy/Collective efficacy (DBCT#10 – DBCT#11)

**Beliefs about Consequences**

Outcome expectancies (DBCT#12 – DBCT#14)

Attitude (DBCT#15)

Values (DBCT#16)

Risk perception (DBCT#17)

**Reinforcement**

Conditioning (DBCT#18 – DBCT#22)

**Goals**

Enjoyment (DBCT#23)

Goals (DBCT#24 – DBCT#25)

Commitment (DBCT#26 – DBCT#28)

**Environmental Context and Resources**

Cue (DBCT#29 – DBCT#30)

**Behavioral Regulation**

Planning (DBCT#31 – DBCT#33)

Proactive planning (DBCT#34 – DBCT#35)

Problem solving (DBCT#36)

Action control (DBCT#37 – DBCT#41)

Behavioral substitution/Counter-conditioning (DBCT#42)

**Social Influences**

Interdependence (DBCT#43)

Modeling (DBCT#44 – DBCT#45)

Disclosure (DBCT#46)

Social control (DBCT#47 – DBCT#49)

Skillful support (DBCT#50 – DBCT#51)

Support function (DBCT#52 – DBCT#58)

Tailored support (DBCT#59)

Support provision skills (DBCT#60 – DBCT#73)

**Dyadic Behavior Change Techniques (DBCTs) for health behavior change**

Please note: Listing of a technique does not imply its effectiveness.

| Domain | | | | |  |  |
| --- | --- | --- | --- | --- | --- | --- |
| No. # | **Dyadic Behavior Change Technique** | | |  |  |  |
|  | **Who?**  **Execution** | **Performs what?**  **Intervention task** | **For whom?**  **Target** | **Example** | **Most proximal mechanism of action** | **Theories of behavior change** |
|  | Who performs the intervention task? | What intervention task is  being performed? | Whom is the intervention task targeted at? | All examples are fictional and represent only one of many possible illustrations. This also applies to the health behavior domain selected in each example. | Theoretical process affecting behavior | Socio-ecological or dyadic theories are listed,  if available. List is non-exhaustive. |
| **Knowledge/Information** (An awareness of the existence of something)^†^ | | | | | | |
| 1a | One partner | **receives information about the health**  **behavior of** | the other partner | Alex receives dietary advice for Billie, who is pregnant. | Information processing |  |
| 1b |  |  | the dyad | Alex receives instructions for muscle-building exercises to do as a couple. |  |  |
| 1c | The dyad |  | one partner | The dyad receives recipes recommended for Alex who has suffered a heart attack. |  |  |
| 1d |  |  | the dyad | The dyad receives information about how to safely use condoms. |  |  |
| 2a | One partner | **shares health information with** | the other partner | Billie is instructed to share information about elevated cancer rates linked to smoking and secondhand smoke with Alex. | Sharing  information | Social integration (Berkman et al., 2000) |
| 2b |  |  | the dyad |  |  |  |
| 2c | The dyad |  | one partner |  |  |  |
| 2d |  |  | the dyad |  |  |  |
|  | | | | | | |
|  | | | | | | |
|  | | | | | | |
|  | | | | | | |
| **Skills** (An ability or proficiency acquired through practice)^D^ | | | | | | |
| 3a  (60a) | One partner | **practices skills for**  **the health behavior of** | the other partner | Alex is encouraged to attend a first-aid course to practice the correct use of an asthma spray in case Billie has an asthma attack. | Behavioral  skills |  |
| 3b |  |  | the dyad | Alex is encouraged to attend a cooking course to practice preparing healthy dinners for them as a dyad. |  |  |
| 3c  (60c) | The dyad |  | one partner | The dyad is instructed to practice together how to perform a breast self-examination that Alex should actively perform afterwards. |  |  |
| 3d |  |  | the dyad | The dyad is guided to jointly practice putting a condom on a condom demonstrator. |  |  |
| 4a  (61a) | One partner | **practices communication skills for the health behavior of** | the other partner | Billie is encouraged to rehearse in the mirror how to talk to Alex about quitting to drink. | Communication  skills | Coping as a communal process (Lyons et al., 1998); Interdependence and communal coping approach (Lewis et al., 2006); Motivational interviewing (Miller & Rollnick, 2012); Nonviolent communication  (Rosenberg, 2015); Supportive interaction (Goldsmith, 1992); Theory of dyadic illness management (Lyons & Lee, 2018) |
| 4b |  |  | the dyad | Billie is encouraged to practice negotiation tactics for safer sex practices. |  |  |
| 4c  (61c) | The dyad |  | one partner | The dyad is guided to practice speaker-listening skills together regarding conversations about Billie’s drinking behavior. |  |  |
| 4d |  |  | the dyad | The dyad is advised to participate in a communication workshop where they as a dyad practice expressing their feelings, concerns, and expectations about safe sex. |  |  |
| Note. The numbers in parentheses indicate cross-references to intervention tasks, which are listed twice in the compendium: once in relation to health behaviors and once in relation to support behaviors. | | | | | | |
| **Social Role and Identity** (A coherent set of behaviors and displayed personal qualities of an individual in a social setting)^†^ | | | | | | |
| 5a | One partner | **takes the**  **perspective on the health behavior change of ^1^** | the other partner | Billie is prompted to try to understand why it is hard for Alex to quit smoking, thinking about barriers and challenges Alex might experience. | Perspective  taking | Theories of stigma and discrimination (Batson & Ahmad, 2009) |
| 5b |  |  | the dyad | Billie is encouraged to understand the stress and impact on their relationship when both of them attempt to quit smoking at the same time. |  |  |
| 5c | The dyad |  | one partner | The dyad is prompted to try to collectively understand how Alex’s life is affected by the need to adhere to a gluten-free diet. |  |  |
| 5d |  |  | the dyad | The dyad is asked to discuss how it will feel for them as a dyad to jointly quit smoking. |  |  |
| 6a | One partner | **recognizes and/or adopts an attitude of being a team for the health behavior change of** | the other partner | Alex is encouraged to see that Billie’s diabetes management is a joint endeavor. | Communal orientation | Coping as a communal process (Lyons et al., 1998); Developmental-contextual model (Berg & Upchurch, 2007); Dyadic coping: a systemic-transactional model (Bodenmann, 1997, 2016); Interdependence and communal coping approach (Lewis et al., 2006); Theory of dyadic illness management (Lyons & Lee, 2018); Social identity theory (Haslam et al., 2018) |
| 6b |  |  | the dyad | Alex is encouraged to recognize that both partners’ overweight is a shared problem that the dyad needs to address together. |  |  |
| 6c | The dyad |  | one partner | The dyad is encouraged to discuss and frame Alex’s hypertension as a shared challenge that they as a dyad need to approach together. |  |  |
| 6d |  |  | the dyad | The dyad is guided to approach their shared goal of increasing physical activity as a team effort that needs collaboration in choosing activities and setting a consistent schedule. |  |  |
|  |  |  |  |  |  |  |
| 7a | One partner | **engages in cooperative action for the health behavior change of** | the other partner | To maximize stimulus control in Alex’s endeavor to lose weight, Billie is encouraged to refrain from eating junk food in Alex’s presence. | Communal action | Coping as a communal process (Lyons et al., 1998); Developmental-contextual Model (Berg & Upchurch, 2007); Dyadic coping: a systemic-transactional model (Bodenmann, 1997, 2016); Interdependence and communal coping approach (Lewis et al., 2006); Theory of dyadic illness management (Lyons & Lee, 2018) |
| 7b |  |  | the dyad | Billie is prompted to coordinate the scheduling of a joint health check-up to address the dyad’s shared unhealthy lifestyle. |  |  |
| 7c | The dyad |  | one partner | The dyad is encouraged to coordinate both partners’ schedules to clear time for Billie to participate in a yoga class. |  |  |
| 7d |  |  | the dyad | The dyad is instructed to divide responsibilities for their joint exercise routine: Billie researches and selects the workouts, while Alex ensures the living space is cleared and ready for exercise. |  |  |
| 8a | One partner | **recognizes and/or adapts norms of** | the other partner | Alex is asked to think about Billie’s eating habits and recognizes Billie’s unspoken rule to always have dessert after dinner. | Norms | Dyadic health influence model (Huelsnitz et al., 2022); Theory of planned behavior (Ajzen, 1991, Fekadu & Kraft, 2002); Social influence (Cialdini & Trost, 1998, Cialdini & Goldstein, 2004); Social identity theory (Haslam et al., 2018) |
| 8b |  |  | the dyad | Alex is encouraged to consider their shared bedtime habits and identifies the norm of watching late-night shows which often leads to reduced sleeping hours. |  |  |
| 8c | The dyad |  | one partner | After discussing how it has become the norm for Alex to work late, the dyad is guided to agree on an evening cut-off time for work to promote better sleep. |  |  |
| 8d |  |  | the dyad | The dyad is encouraged to adapt their norm of watching TV after dinner by deciding to cultivate a new habit of taking short walks after meals. |  |  |
| 9a | One partner | **takes on the role of a coach* for** | the other partner | Billie is instructed to assist, teach and motivate Alex while exercising. | Roles | Attachment, roles, and social support (Kahn & Antonucci, 1980) |
| 9b |  |  | the dyad |  |  |  |
| 9c | The dyad |  | one partner |  |  |  |
| 9d |  |  | the dyad |  |  |  |
| **Beliefs about Capabilities** (Acceptance of the truth, reality or validity about an ability, talent or facility that a person can put to constructive use)^†^ | | | | | | |
| 10a  (62a) | One partner | **reviews past mastery experiences of** | the other partner | Alex is instructed to recall instances when Billie successfully met or exceeded the weekly recommendation of 150 minutes of moderate to vigorous physical activity. | Self-efficacy/ Dyadic efficacy/ Collective efficacy | Dyadic efficacy (Sterba et al., 2017); Dyadic coping: a systemic-transactional model (Bodenmann, 1997, 2016);  Social cognitive theory (Bandura, 2001, McAlister et al., 2008) |
| 10b |  |  | the dyad | Alex is prompted to recollect successful changes in their safer sex habits. |  |  |
| 10c  (62c) | The dyad |  | one partner | The dyad is encouraged to recollect together what Alex achieved so far in terms of reducing daily sedentary time. |  |  |
| 10d |  |  | the dyad | The dyad is invited to reflect together on times when they successfully managed to jointly cook healthy meals at home. |  |  |
| 11a | One partner | **reviews skills**  **of** | the other partner | Billie is guided to reflect on the skills Alex learned in therapy to manage cravings for alcohol. |  |  |
| 11b |  |  | the dyad | Billie is instructed to list the skills the dyad possesses to prevent HIV transmission within the dyad. |  |  |
| 11c | The dyad |  | one partner | The dyad is prompted to list the skills Billie possesses including breathing exercises and self-instructions to resist smoking. |  |  |
| 11d |  |  | the dyad | The dyad is encouraged to review the skills they have as a dyad to engage in more physical activity such as playing well together in a volleyball team. |  |  |
| Note. The numbers in parentheses indicate cross-references to intervention tasks, which are listed twice in the compendium: once in relation to health behaviors and once in relation to support behaviors. | | | | | | |
| **Beliefs about Consequences** (Acceptance of the truth, reality, or validity about outcomes of a behavior in a given situation)^†^ | | | | | | |
| 12a | One partner | **weighs the pros and cons of the health behavior for ^2^** | the other partner | Alex is asked to weigh Billie’s advantages and disadvantages of cycling to work. | Outcome expectancies | Coping as a communal process (Lyons et al., 1998); Dyadic health influence model (Huelsnitz et al., 2022);  Interdependence and communal coping approach (Lewis et al., 2006); Social cognitive theory (Bandura, 2001; McAlister et al., 2008) |
| 12b |  |  | the dyad | Alex is instructed to weigh the pros and cons of the various safer sex methods they as a dyad could use. |  |  |
| 12c | The dyad |  | one partner | The dyad is guided to jointly weigh the reasons for and against a gastric band for Alex. |  |  |
| 12d |  |  | the dyad | The dyad is encouraged to jointly weigh the advantages and disadvantages of adopting a vegetarian diet for their shared meals. |  |  |
| 13a | One partner | **recognizes health consequences for** | the other partner | Reading an information brochure on the risks of skin cancer, Billie is prompted to recognize how Alex’s habit of using sunscreen decreases Alex’s risk of skin cancer. |  |  |
| 13b |  |  | the dyad | Billie is prompted to read about the risk of STD transmission within the dyad to recognize that condom use is usually the recommended safer sex practice. |  |  |
| 13c | The dyad |  | one partner | The dyad is guided to understand how adopting a healthier diet can lead to a significant reduction in Billie’s elevated cholesterol levels. |  |  |
| 13d |  |  | the dyad | The dyad is encouraged to reflect on the long-term health benefits of their decision to regularly hike together, such as enhanced cardiovascular health and lower stress levels. |  |  |
|  |  |  |  |  |  |  |
| 14a | One partner | **recognizes relationship consequences from health behavior of** | the other partner | Alex is prompted to reflect on how Billie’s adopting a healthier lifestyle could lead to a longer life and them spending many more happy years together. | Outcome expectancies | Coping as a communal process (Lyons et al., 1998); Dyadic health influence model (Huelsnitz et al., 2022);  Interdependence and communal coping approach (Lewis et al., 2006); Social cognitive theory (Bandura, 2001; McAlister et al., 2008) |
| 14b |  |  | the dyad | Alex is prompted to recognize that the time both partners spend on physical activity can be viewed as quality time spent together which can also enhance the dyad’s relationship quality. |  |  |
| 14c | The dyad |  | one partner | The dyad is encouraged to reflect on how Alex’s habit to go out drinking with friends each weekend also leads to feelings of neglect and distance in Billie. |  |  |
| 14d |  |  | the dyad | The dyad is advised to discuss how their drinking behavior during joint dinners makes them more prone to relationship conflicts. |  |  |
| 15a | One partner | **recognizes and/or changes attitude towards the health behavior of** | the other partner | Billie is guided to reflect on the benefits of Alex’s plant-based diet and to adopt a more positive attitude towards Alex’s diet. | Attitude | Dyadic health influence model (Huelsnitz et al., 2022); Social integration (Berkman et al., 2000); Theory of planned behavior (Ajzen, 1991; Fekadu & Kraft, 2002); Social identity theory (Haslam et al., 2018) |
| 15b |  |  | the dyad | Billie is encouraged to reflect on the importance and benefits of the dyad’s consistent condom use and to adopt a more positive attitude. |  |  |
| 15c | The dyad |  | one partner | The dyad is prompted to recognize that being physically active actually could be fun for Billie even though Billie would not have thought so before. |  |  |
| 15d |  |  | the dyad | The dyad is encouraged to re-evaluate and change their initial skepticism towards their joint decision to consistently use condoms, recognizing the mutual benefits for their health and relationship. |  |  |
|  |  |  |  |  |  |  |
| 16a | One partner | **recognizes and/or changes values towards the health behavior of** | the other partner | Billie is guided to recognize the ethical and environmental values of Alex's plant-based diet. | Values | Social identity theory (Haslam et al., 2018) |
| 16b |  |  | the dyad | Billie is encouraged to recognize that using condoms reflects the dyad’s strong valuation of health. |  |  |
| 16c | The dyad |  | one partner | To facilitate Alex’s goal to commute to work by bike every day, the dyad is prompted to recognize their shared value of prioritizing vitality and health. |  |  |
| 16d |  |  | the dyad | The dyad is encouraged to view their regular check-ups and screenings as an expression of prioritizing their health and longevity together. |  |  |
| 17a | One partner | **recognizes risk for ^3^** | the other partner | Alex is encouraged to reflect on the risks Alex’s smoking poses to Billie due to secondhand exposure. | Risk  perception | Coping as a communal process (Lyons et al., 1998); Developmental-contextual model (Berg & Upchurch, 2007); Dyadic health influence model (Huelsnitz et al., 2022);  Interdependence and communal coping approach (Lewis et al., 2006); Theory of dyadic illness management (Lyons & Lee, 2018) |
| 17b |  |  | the dyad | Alex is advised to consider the risks of the dyad’s unsafe sexual practices, including a heightened risk of HIV transmission and other sexually transmitted infections. |  |  |
| 17c | The dyad |  | one partner | The dyad is prompted to acknowledge that Alex’s sedentary lifestyle, including long hours of watching TV, could increase the risk of developing heart disease. |  |  |
| 17d |  |  | the dyad | The HIV concordant dyad is promoted to recognize the risks of drug resistance and potential super-infection without consistent condom use. |  |  |
|  |  |  |  |  |  |  |
| **Reinforcement** (Increasing the probability of a response by arranging a dependent relationship, or contingency, between the response and a given stimulus)^†^ | | | | | | |
| 18a | One partner | **provides reward (social/material/unspecified) to ^4^** | the other partner | Billie is encouraged to compliment Alex's choice of a healthy meal over fast food. | Conditioning | Behavioral couples therapy (Epstein & Baucom, 2002; O’Farrell & Fals-Stewart, 2006; O’Farrell & Schein, 2011);  Social cognitive theory (Bandura, 2001; McAlister et al., 2008) |
| 18b |  |  | the dyad | Billie is prompted to provide a reward to them as a dyad and decides to buy concert tickets for them after both partners successfully reached their goal of running a half-marathon together. |  |  |
| 18c | The dyad |  | one partner | Being encouraged to provide a reward, the dyad celebrates Billie’s reaching the target weight by buying Billie a new outfit. |  |  |
| 18d |  |  | the dyad | Being advised to reward themselves, the dyad books a weekend getaway after successfully quitting to smoke for six months. |  |  |
| 19a | One partner | **provides reward (social/material/unspecified) for progress/effort of** | the other partner | Alex is guided to express pride in Billie's achievement of successfully losing the first 2kg (4.4lb) towards the targeted 15kg (33.1lb) weight loss. |  |  |
| 19b |  |  | the dyad | Being encouraged to reward themselves as a couple, Alex books a weekend trip to celebrate that both partners are trying to reduce the number of cigarettes smoked per week. |  |  |
| 19c | The dyad |  | one partner | Being prompted to reward that Alex started taking the bike instead of the car more often, the dyad spends an exclusive day together doing activities Alex loves, as a form of social recognition. |  |  |
| 19d |  |  | the dyad | Being encouraged to reward their progress, the dyad rewards themselves with a new blender for making healthier smoothies after successfully reducing processed sugars from their joint meals for a month. |  |  |
|  |  |  |  |  |  |  |
| 20a | One partner | **removes reward (social/material/unspecified) in case of failure of ^5^** | the other partner | Being encouraged to remove rewards in case of failure, Billie withdraws from demonstrating understanding and compassion when Alex fails to adhere to the goal of abstaining from alcohol. | Conditioning | Behavioral couples therapy (Epstein & Baucom, 2002; O’Farrell & Fals-Stewart, 2006; O’Farrell & Schein, 2011);  Social cognitive theory (Bandura, 2001; McAlister et al., 2008) |
| 20b |  |  | the dyad | Being instructed to remove rewards for them as a couple in case of failure, Billie cancels the dyad’s planned weekend getaway after they missed their joint exercise sessions for several days. |  |  |
| 20c | The dyad |  | one partner | The dyad is prompted to reconsider their joint reward of a movie marathon after Billie misses their workout sessions. |  |  |
| 20d |  |  | the dyad | After setting up a reward system where they would watch their favorite show together for achieving 10,000 daily steps, the dyad is suggested to skip the show on days they don't reach their goal. |  |  |
| 21a | One partner | **identifies and/or changes own role in maintaining the risk behavior of** | the other partner | Alex is guided to recognize that Alex’s tolerance of smoking indoors contributes to Billie’s continued smoking behavior. |  |  |
| 21b |  |  | the dyad | Alex is prompted to acknowledge that Alex’s passion for watching movies contributes to the dyad’s joint sedentary lifestyle and proposes the idea of joining a local sports league or dance class together. |  |  |
| 21c | The dyad |  | one partner | The dyad is instructed to analyze how their habit of ordering takeout contributes to Alex’s tendency to overeat and consume unhealthy foods. |  |  |
| 21d |  |  | the dyad | The dyad is encouraged to identify that their habit of keeping their home stocked with alcohol contributes to their joint tendency to drink excessively. |  |  |
|  |  |  |  |  |  |  |
| 22a | One partner | **sets a (social/material/unspecified) incentive for ^6^** | the other partner | Being instructed to set an incentive, Billie offers Alex a day at the spa if Alex goes to the yearly check-up. | Conditioning | Behavioral couples therapy (Epstein & Baucom, 2002; O’Farrell & Fals-Stewart, 2006; O’Farrell & Schein, 2011);  Social cognitive theory (Bandura, 2001; McAlister et al., 2008) |
| 22b |  |  | the dyad | Billie is instructed to set an incentive, and as a motivational gesture brings home vacation brochures and plans a potential trip if both partners achieve their weight loss goals. |  |  |
| 22c | The dyad |  | one partner | Being encouraged to set an incentive, the dyad agrees that after attending the cardiac sports group, Billie will get a day at the spa as a treat. |  |  |
| 22d |  |  | the dyad | To encourage healthier habits, the dyad is guided to set a rule: if they both replace evening snacks with fruits for two weeks straight, they will have a themed movie night with all their favorite films. |  |  |
| **Goals** (Mental representations of outcomes or end states that an individual wants to achieve)^†^ | | | | | | |
| 23a | One partner | **identifies enjoyable behaviors for** | the other partner | Alex is guided to identify enjoyable ways to engage in physical activity that Billie relished in the past to address Billie’s waning enthusiasm. | Enjoyment | Companionship (Rook, 1987, 2015) |
| 23b |  |  | the dyad | To find shared activities, Alex is instructed to identify and list physical activities they as a dyad found enjoyable in the past. |  |  |
| 23c | The dyad |  | one partner | The dyad is prompted to identify and discuss healthy dishes Alex has enjoyed cooking previously. |  |  |
| 23d |  |  | the dyad | The dyad is encouraged to identify and discuss safer sex methods they as a dyad find enjoyable or appealing. |  |  |
|  |  |  |  |  |  |  |
| 24a | One partner | **selects health**  **behavior for** | the other partner | Billie is guided to select appropriate mindfulness exercises for Alex to reduce distress. | Goals | Coping as a communal process (Lyons et al., 1998);  Interdependence and communal coping approach (Lewis et al., 2006); Social cognitive theory (Bandura, 2001, McAlister et al. 2008); Theory of dyadic illness management (Lyons & Lee, 2018); Transactive goal dynamics theory (Fitzsimons et al., 2015) |
| 24b |  |  | the dyad | Being instructed to determine how to eat healthier as a dyad, Billie decides for them to increase intake of fruits and vegetables to improve their joint diet in the upcoming week. |  |  |
| 24c | The dyad |  | one partner | Being encouraged to select a health behavior to offset the sedentary nature of Billie's job, the dyad selects yoga for Billie to boost flexibility and relaxation. |  |  |
| 24d |  |  | the dyad | The dyad is directed to select a health behavior for them as a dyad from a list, opting for a specific safer sex method. |  |  |
| 25a  (64a) | One partner | **sets a goal**  **for ^7^** | the other partner | Alex is instructed to set the goal for Billie to engage in daily fall prevention exercises. |  |  |
| 25b |  |  | the dyad | Alex is advised to set the goal for them as a dyad to consistently use sunblock during their vacation. |  |  |
| 25c  (64c) | The dyad |  | one partner | Being encouraged to set a goal for Alex, the dyad decides on the goal to start flossing every day. |  |  |
| 25d |  |  | the dyad | Being instructed to set a goal for them as a dyad, they set the joint goal of achieving a combined total of 15,000 daily steps. |  |  |
| Note. The numbers in parentheses indicate cross-references to intervention tasks, which are listed twice in the compendium: once in relation to health behaviors and once in relation to support behaviors | | | | | | |
|  |  |  |  |  |  |  |
|  |  |  |  |  |  |  |
|  |  |  |  |  |  |  |
| 26 (65a) | One partner | **commits to**  **a goal of ^8^** | the other partner | Billie is guided to commit to Alex’s goal to lose weight. | Commitment | Behavioral couples therapy (Epstein & Baucom, 2002; O’Farrell & Fals-Stewart, 2006; O’Farrell & Schein, 2011);  Coping as a communal process (Lyons et al., 1998); Interdepen-  dence and communal coping approach (Lewis et al., 2006); Theory of dyadic illness mange- men (Lyons & Lee, 2018); Transactive goal dynamic theory (Fitzsimons et al., 2015) |
| 26b |  |  | the dyad | Billie is encouraged to commit to the dyad's goal to refrain from purchasing sweets during grocery shopping. |  |  |
| 26c  (65c) | The dyad |  | one partner | Acknowledging Billie's weight loss aspiration, the dyad is instructed to commit to the goal of preparing and consuming healthier meals. |  |  |
| 26d |  |  | the dyad | The dyad is prompted to dedicate themselves to the goal of consistently using protection for their safer intimacy. |  |  |
| 27a | One partner | **reviews and/or adapts goals for ^9^** | the other partner | Alex is guided to review Billie's goal of running 10 km (6.2 miles) weekly and, after observing improved stamina, suggests increasing the target to 15 km (9.3 miles). |  |  |
| 27b |  |  | the dyad | Alex is instructed to review the goals for Alex and Billie’s joint exercise sessions, reflecting on the challenges and ease, and then to adjust the objectives based on this assessment. |  |  |
| 27c | The dyad |  | one partner | The dyad is prompted to review the weight loss goal set for Alex the previous year and to discuss potential revisions for the next year. |  |  |
| 27d |  |  | the dyad | The dyad is encouraged to review their goal of joint meditation, considering whether to maintain or modify the frequency, or to explore a different relaxation technique. |  |  |
| 28a  (66a) | One partner | **makes a**  **contract for ^10^** | the other partner |  |  |  |
| 28b |  |  | the dyad |  |  |  |
| 28c  (66c) | The dyad |  | one partner | The dyad is prompted to create and sign a contract committing Billie to quit alcohol. |  |  |
| 28d |  |  | the dyad | The dyad is encouraged to make a contract ensuring that both partners adhere to their joint goal of quitting to smoke. |  |  |
| Note. The numbers in parentheses indicate cross-references to intervention tasks, which are listed twice in the compendium: once in relation to health behaviors and in relation to support behaviors. | | | | | | |
| **Environmental Context and Resources** (Any circumstance of a person’s situation or environment that discourages or encourages the development of skills and abilities, independence, social competence and adaptive behavior)^†^ | | | | | | |
| 29a  (67a) | One partner | **controls (including setting and removing) cues for ^11^** | the other partner | Alex is directed to control cues by removing the candy bowl from the living room table, a known trigger for Billie's snacking habit. | Cue | Behavioral couples therapy (Epstein & Baucom, 2002; O’Farrell & Fals-Stewart, 2006; O’Farrell & Schein, 2011) |
| 29b |  |  | the dyad | Alex is instructed to put vegetables instead of potato chips or crisps on the coffee table to reduce joint unhealthy snacking when watching TV together. |  |  |
| 29c | The dyad |  | one partner | The dyad is prompted to control cues by removing ashtrays and lighters from their living space, assisting Alex to quit smoking. |  |  |
| 29d |  |  | the dyad | The dyad is encouraged to control cues by clearing out all alcoholic beverages from their residence, aiding their mutual goal to abstain from alcohol. |  |  |
| 30a | One partner | **identifies and/or changes own behavior that serves as a trigger for** | the other partner | Billie is asked to reflect on which of Billie’s behaviors might serve as a trigger for Alex’s snacking behavior, and to change these triggering behaviors. |  |  |
| 30b |  |  | the dyad | Billie is prompted to identify own negative communication patterns that lead the dyad to drink alcohol, and to consider how to improve them. |  |  |
| 30c | The dyad |  | one partner | The dyad is instructed to list what they do as a dyad that triggers the urge to smoke in Billie. |  |  |
| 30d |  |  | the dyad | The dyad is instructed to make a list of joint activities that lead them to use substances, and to attempt to change at least one of these activities. |  |  |
| Note. The numbers in parentheses indicate cross-references to intervention tasks, which are listed twice in the compendium: once in relation to health behaviors and once in relation to support behaviors. | | | | | | |
| **Behavioral Regulation** (Anything aimed at managing or changing objectively observed or measured actions)^†^ | | | | | | |
| 31a  (69a) | One partner | **plans for ^12^** | the other partner | Alex is instructed to plan a detailed meal schedule with healthy options for Billie to cut down on sugar. | Planning | Collaborative implementation intentions (Prestwich et al., 2005); Dyadic planning (Burkert et al., 2011) |
| 31b |  |  | the dyad | Alex is encouraged to plan where and when they as a dyad could go on regular bicycle tours together. |  |  |
| 31c  (69c) | The dyad |  | one partner | The dyad is invited to write down plans specifying when and where Alex can perform muscle strength exercises. |  |  |
| 31d |  |  | the dyad | The dyad is prompted to collaboratively plan a weekly meal prep routine, ensuring their meals align with their shared objective of consuming more whole foods. |  |  |
| 32a | One partner | **reviews and/or adapts plans for** | the other partner | Billie is prompted to review Alex's plan for an exercise routine and to consider potential amendments. |  |  |
| 32b |  |  | the dyad | Billie is guided to review the dyad's initial plans for safer sex methods and adjust them based on the dyad's evolving needs. |  |  |
| 32c | The dyad |  | one partner | The dyad is encouraged to review Billie's plan to eat more fiber and to discuss alternative actions if needed. |  |  |
| 32d |  |  | the dyad | The dyad is instructed to review their weekly outdoor exercise plans and to come up with alternative approaches for the winter. |  |  |
| Note. The numbers in parentheses indicate cross-references to intervention tasks, which are listed twice in the compendium: once in relation to health behaviors and once in relation to support behaviors. | | | | | | |
| 33a | One partner | **creates a**  **coping plan for** | the other partner | Alex is directed to propose a coping plan for Billie in case Billie deviates from the original plan of abstaining from alcohol during dinner by devising behavioral strategies to help maintain sobriety. | Planning | Collaborative implementation intentions (Prestwich et al., 2005); Dyadic planning (Burkert et al., 2011) |
| 33b |  |  | the dyad | Alex is guided to suggest a coping plan for the event that the dyad faces challenges in adhering to their initial plan to use alternative safer sex methods by strategizing joint coping mechanisms. |  |  |
| 33c | The dyad |  | one partner | The dyad is prompted to discuss challenging upcoming events and to plan for Alex how to stick to the original plan to avoid highly processed foods and adhere to a healthy diet. |  |  |
| 33d |  |  | the dyad | The dyad is encouraged to plan what they would do to maintain their abstinence from substances if their original plan to avoid high-risk events failed. |  |  |
| 34a | One partner | **Identifies and/or implements facilitators for behavioral performance of** | the other partner | Billie is directed to pinpoint strategies that would help Alex to consistently use a helmet when cycling. | Proactive planning |  |
| 34b |  |  | the dyad | In light of the dyad’s shared objective for daily activity, Billie is prompted to identify local areas that would encourage them as a dyad to engage in exercise together in the evening. |  |  |
| 34c | The dyad |  | one partner | The dyad is guided to identify which types of motivating music or podcasts help Billie stick to the jogging routine. |  |  |
| 34d |  |  | the dyad | The dyad is prompted to discuss settings that facilitate their joint attempt to quit smoking. |  |  |
|  | | | | | | |
| 35a | One partner | **identifies and/or performs preparatory behaviors for** | the other partner | Alex is prompted to list preparatory steps for Billie to begin exercising, including purchasing appropriate footwear and finding a suitable jogging path. | Proactive planning |  |
| 35b |  |  | the dyad | Alex is instructed to make a checklist of what they as a dyad have to do before they cook a healthy dinner together, such as deciding on a recipe and buying ingredients. |  |  |
| 35c | The dyad |  | one partner | The dyad is encouraged to discuss initial steps to be taken for Alex to free up time for an evening walk. |  |  |
| 35d |  |  | the dyad | The dyad is asked to determine responsibilities regarding the purchase of condoms for practicing safer intimacy. |  |  |
| 36a  (70a) | One partner | **identifies barriers with identification of solutions (problem solving) for ^13^** | the other partner | Billie is guided to identify barriers that deter Alex from adhering to the prescribed medication schedule and brainstorm potential solutions. | Problem  solving | Coping as a communal process (Lyons et al., 1998);  Developmental-contextual model (Berg & Upchurch, 2007);  Interdependence and communal coping approach (Lewis et al., 2006);  Social support and marital coping (Revenson, 1994); Theory of dyadic illness management (Lyons & Lee, 2018) |
| 36b |  |  | the dyad | Billie is prompted to reflect on challenges they as a dyad experienced in the past that hindered them to practice safer sex to prevent HIV transmission and to write down possible solutions. |  |  |
| 36c  (70c) | The dyad |  | one partner | The dyad is encouraged to identify challenges Billie faces in following the plan to reduce sugar intake and collaboratively think of potential solutions. |  |  |
| 36d |  |  | the dyad | The dyad is directed to discuss barriers preventing them from their goal of having joint gym sessions and devise solutions. |  |  |
| 37a | One partner | **prompts awareness of goals of** | the other partner | Alex is prompted to remind Billie of the goal of applying sunblock daily. | Action control | Dyadic action control (Scholz & Berli, 2014; Berli et al., 2016) |
| 37b |  |  | the dyad | Alex is advised to put a post-it on the fridge with a reminder for them as a dyad to reduce snacking after dinner. |  |  |
| 37c | The dyad |  | one partner | The dyad is encouraged to prompt Alex's goal of reaching 10,000 steps daily by putting a sticker on the back of Alex’s smartphone. |  |  |
| 37d |  |  | the dyad | The dyad is advised to put a reminder on their bathroom mirror, emphasizing their mutual goal of daily flossing. |  |  |
| Note. The numbers in parentheses indicate cross-references to intervention tasks, which are listed twice in the compendium: once in relation to health behaviors and once in relation to support behaviors. | | | | | | |
| 38a | One partner | **prompts**  **self-monitoring of** | the other partner | Billie is directed to prompt self-monitoring by inquiring about Alex's progress on Alex’s weekly physical activity goals. | Action control | Dyadic action control (Scholz & Berli, 2014; Berli et al., 2016) |
| 38b |  |  | the dyad | Billie is instructed to remind them as a dyad to enter the number of cigarettes smoked each day in a smoking cessation app. |  |  |
| 38c | The dyad |  | one partner | To prompt self-monitoring, the dyad is encouraged to install a water intake tracker on the fridge, allowing Billie to mark each glass of water consumed. |  |  |
| 38d |  |  | the dyad | The dyad is guided to establish a shared calendar, prompting self-monitoring of their collective daily caloric intake. |  |  |
| 39a | One partner | **prompts goal-related action, if goal is not met, for** | the other partner | Alex is advised to suggest to Billie the use of a fitness tracker when Billie’s adherence to the physical activity goal is not sufficient. |  |  |
| 39b |  |  | the dyad | Alex is advised to replace the afternoon cookie snack with some chopped vegetables to achieve their shared goal of reducing sugar intake that day. |  |  |
| 39c | The dyad |  | one partner | The dyad is guided to establish cues such as leaving the bike in front of the door, when noticing that Alex’s goal to be sufficiently physically active has not been met yet. |  |  |
| 39d |  |  | the dyad | The dyad is instructed to store condoms next to the bed to prompt them to more consistently engage in their agreed upon safer sex behaviors. |  |  |
| 40a  (71a) | One partner | **monitors ^14^** | the other partner | Billie is directed to monitor and record Alex's blood pressure. |  |  |
| 40b |  |  | the dyad | Billie is asked to keep track of their consistent use of condoms. |  |  |
| 40c  (71c) | The dyad |  | one partner | The dyad is encouraged to collaboratively monitor and note down Billie's daily water intake. |  |  |
| 40d |  |  | the dyad | The dyad is guided to jointly record lapses in their new shared dietary plan. |  |  |
| Note. The numbers in parentheses indicate cross-references to intervention tasks, which are listed twice in the compendium: once in relation to health behaviors and once in relation to support behaviors. | | | | | | |
| 41a  (72a) | One partner | **reviews the health**  **behavior of** | the other partner | Alex is advised to review Billie's weight loss actions, including Billie’s diet and exercise behaviors. | Action control | Dyadic action control (Scholz & Berli, 2014; Berli et al., 2016) |
| 41b |  |  | the dyad | Alex is instructed to analyze the foods they as a dyad consume during their shared meals. |  |  |
| 41c  (72c) | The dyad |  | one partner | The dyad is prompted to assess Alex’s medication adherence during the past month. |  |  |
| 41d |  |  | the dyad | The dyad is encouraged to reflect on the safer sex methods they've practiced in the past and discuss their experiences. |  |  |
| 42a | One partner | **identifies behavioral substitution for ^15^** | the other partner | Billie is instructed to list alternative activities when encountering triggers for drinking. | Behavioral substitution/ Counter-conditioning | Behavioral couples therapy (Epstein & Baucom, 2002; O’Farrell & Fals-Stewart, 2006; O’Farrell & Schein, 2011) |
| 42b |  |  | the dyad | Billie is prompted to suggest replacing evening TV sessions with joint walks. |  |  |
| 42c | The dyad |  | one partner | The dyad is guided to identify non-alcoholic beverage alternatives during their joint dinners for Billie aiming to cut down on alcohol. |  |  |
| 42d |  |  | the dyad | The dyad is encouraged to think about healthier substitutes for their late-night ice cream treats, such as fruit salads. |  |  |
| **Social Influences** (Those interpersonal processes that can cause individuals to change their thoughts, feelings, or behaviors)^†^ | | | | | | |
| 43a | One partner | **recognizes direct and indirect impact of** | the other partner | Alex is instructed to recognize that Alex adopted Billie’s sleeping habits. | Inter-dependence | Interdependence theory (Rusbult & van Lange, 2003) |
| 43b |  |  | the dyad | Alex is prompted to recognize that their joint ritual to start their mornings with a shared cigarette is making their joint quit attempt harder. |  |  |
| 43c | The dyad |  | one partner | The dyad is prompted to recognize the positive influence of Alex's habit of preparing fruits in the morning, leading both to make healthier eating choices. |  |  |
| 43d |  |  | the dyad | The dyad is directed to reflect on the detrimental health effects of their favorite activity to do as a dyad, that is their joint sedentary behavior in front of the television. |  |  |
| Note. The numbers in parentheses indicate cross-references to intervention tasks, which are listed twice in the compendium: once in relation to health behaviors and once in relation to support behaviors. | | | | | | |
| 44a | One partner | **demonstrates the health behavior**  **to ^16^** | the other partner | Billie is instructed to demonstrate a knee-friendly running technique to Alex. | Modeling | Social cognitive theory (Bandura, 2001; McAlister et al., 2008); Companionship (Rook, 1987, 2015) |
| 44b |  |  | the dyad |  |  |  |
| 44c | The dyad |  | one partner |  |  |  |
| 44d |  |  | the dyad |  |  |  |
| 45a | One partner | **performs health behavior of ^17^** | the other partner | Alex is encouraged to join Billie’s exercise class. |  |  |
| 45b |  |  | the dyad | Alex is instructed to adhere to the HIV medication regime to encourage safer sex of the dyad. |  |  |
| 45c | The dyad |  | one partner | Following Alex’s heart attack, the dyad is guided to undertake cardiovascular-friendly exercises together. |  |  |
| 45d |  |  | the dyad | The dyad is encouraged to attend a couple’s dance class together. |  |  |
| 46a | One partner | **shares thoughts and feelings towards the health behavior with** | the other partner | Billie is encouraged to share feelings with Alex about not being able to join the colleagues for smoke breaks. | Disclosure | Thriving through relationships (Feeney & Collins, 2015) |
| 46b |  |  | the dyad |  |  |  |
| 46c | The dyad |  | one partner |  |  |  |
| 46d |  |  | the dyad |  |  |  |
|  |  |  |  |  |  |  |
| 47a | One partner | **regulates the health behavior**  **of ^18^** | the other partner | Billie is assigned the responsibility of managing Alex's medication, including organizing the pill dispenser and strictly enforcing the timing of medication intake. | Social  control | Dual effect model of social control (Hughes & Gove, 1981; Lewis & Rook, 1999); Social integration (Berkman et al., 2000) |
| 47b |  |  | the dyad | Noticing their joint tendency to skip breakfast, Billie takes the initiative to prepare a healthy breakfast for both, emphasizing the importance of a nutritious start in the day. |  |  |
| 47c | The dyad |  | one partner |  |  |  |
| 47d |  |  | the dyad |  |  |  |
| 48a | One partner | **communicates persuasively about the health behavior change of ^19^** | the other partner | Concerned about Billie's health, Alex is encouraged to persuade Billie about the importance of regular medical check-ups, sharing stories of early disease detection and the difference it made for friends or family members. |  |  |
| 48b |  |  | the dyad |  |  |  |
| 48c | The dyad |  | one partner |  |  |  |
| 48d |  |  | the dyad |  |  |  |
| 49a | One partner | **sets out negative consequences**  **for ^20^** | the other partner | Billie is prompted to assign additional household tasks to Alex if Alex fails to reach the goal of walking 10,000 steps daily. |  |  |
| 49b |  |  | the dyad | Billie is prompted to decide on a negative consequence such as not watching a movie together in case the dyad lapses in their joint exercise routine. |  |  |
| 49c | The dyad |  | one partner | The dyad is guided to set a rule that if Billie skips the morning relaxation routine, Billie will have to take out the trash for the entire next month. |  |  |
| 49d |  |  | the dyad | The dyad is encouraged to commit to a pact that missing their joint exercise sessions twice a week results in the obligation to run an extra mile for each missed hour. |  |  |
| 50a | One partner | **identifies potential support sources/social network for** | the other partner | Billie is instructed to make a list of close others who would make home visits to check on Alex after surgery. | Skillful  support | Need support (Niemiec et al., 2014); Relationships motivation theory (Deci & Ryan, 2014, Ryan & Deci, 2000); Responsiveness (Reis, 2014); Skilled support (Rafaeli & Gleason, 2009);  Social cognitive theory (Bandura, 2001, McAlister et al., 2008) |
| 50b |  |  | the dyad | Billie is directed to make a list of people who would be willing to listen to their joint struggles in adhering to a healthy diet. |  |  |
| 50c | The dyad |  | one partner | The dyad is invited to discuss potential individuals who could assist Billie with transportation to the hospital when needed. |  |  |
| 50d |  |  | the dyad | The dyad is advised to discuss who might assist them by providing advice on how to engage in more physical activity during a busy work week. |  |  |
| 51a | One partner | **receives education for supporting** | the other partner | Alex is instructed on how to offer effective support to Billie during chemotherapy, focusing on potential side effects and strategies to alleviate them. |  |  |
| 51b |  |  | the dyad |  |  |  |
| 51c | The dyad |  | one partner | The dyad participates in a workshop to learn techniques on how to provide skillful support to Alex, specifically focusing on assisting with daily blood pressure measurements. |  |  |
| 51d |  |  | the dyad |  |  |  |
|  |  |  |  |  |  |  |
| 52a | One partner | **provides informational support to** | the other partner | To assist Alex in managing Alex’s dermatitis effectively, Billie is encouraged to inform Alex about treatment options. | Support  function | Social support, stress and the buffering hypothesis, (Cohen & McKay, 1984); Developmental-contextual model (Berg & Upchurch, 2007); Dyadic coping: systemic-transactional model of dyadic coping (Bodenmann, 1997, 2016); Need support (Niemiec et al., 2014); Relationships motivation theory (Deci & Ryan, 2014, Ryan & Deci, 2000); Social support and health (Holt-Lunstad & Uchino, 2015); Thriving through social relationships (Feeney & Collins, 2015) |
| 52b |  |  | the dyad |  |  |  |
| 52c | The dyad |  | one partner |  |  |  |
| 52d |  |  | the dyad |  |  |  |
| 53a | One partner | **provides instrumental support to ^21^** | the other partner | To support Billie’s pursuit to lose weight, Alex is encouraged to cook a healthy dinner for Billie. |  |  |
| 53b |  |  | the dyad |  |  |  |
| 53c | The dyad |  | one partner |  |  |  |
| 53d |  |  | the dyad |  |  |  |
| 54a | One partner | **provides emotional support to ^22^** | the other partner | To support Alex’s goal of being more physically active, Billie is prompted to respond empathetically and supportively when Alex shares experiences, such as those from a gym class. |  |  |
| 54b |  |  | the dyad |  |  |  |
| 54c | The dyad |  | one partner |  |  |  |
| 54d |  |  | the dyad |  |  |  |
| 55a | One partner | **provides autonomy support to** | the other partner | Alex is prompted to provide a supportive environment, offering Billie the freedom to choose the type of physical activity Billie enjoys most, rather than insisting on a particular exercise. | Support  function | Social support, stress and the buffering hypothesis, (Cohen & McKay, 1984); Developmental-contextual model (Berg & Upchurch, 2007); Dyadic coping: systemic-transactional model of dyadic coping (Bodenmann, 1997, 2016); Need support (Niemiec et al., 2014); Relationships motivation theory (Deci & Ryan, 2014, Ryan & Deci, 2000); Social support and health (Holt-Lunstad & Uchino, 2015); Thriving through social relationships (Feeney & Collins, 2015) |
| 55b |  |  | the dyad |  |  |  |
| 55c | The dyad |  | one partner |  |  |  |
| 55d |  |  | the dyad |  |  |  |
| 56a | One partner | **provides support (unspecified) to ^23^** | the other partner | Billie is encouraged to offer support to Alex in the pursuit of eating healthier. |  |  |
| 56b |  |  | the dyad |  |  |  |
| 56c | The dyad |  | one partner |  |  |  |
| 56d |  |  | the dyad |  |  |  |
| 57a | One partner | **seeks social support from ^24^** | the other partner | Alex is encouraged to ask Billie for support by asking to take over some household tasks to have more time to go running. |  |  |
| 57b |  |  | the dyad |  |  |  |
| 57c | The dyad |  | one partner |  |  |  |
| 57d |  |  | the dyad |  |  |  |
|  |  |  |  |  |  |  |
| 58a | One partner | **increases receptiveness for social support from** | the other partner | Billie is guided to become more open to Alex's emotional support provision, embracing opportunities to converse and express emotions during challenging times. | Support  function | Social support, stress and the buffering hypothesis, (Cohen & McKay, 1984); Developmental-contextual model (Berg & Upchurch, 2007); Dyadic coping: systemic-transactional model of dyadic coping (Bodenmann, 1997, 2016); Need support (Niemiec et al., 2014); Relationships motivation theory (Deci & Ryan, 2014, Ryan & Deci, 2000); Social support and health (Holt-Lunstad & Uchino, 2015); Thriving through social relationships (Feeney & Collins, 2015) |
| 58b |  |  | the dyad |  |  |  |
| 58c | The dyad |  | one partner |  |  |  |
| 58d |  |  | the dyad |  |  |  |
| 59a | One partner | **identifies support**  **needs of** | the other partner | Billie is asked to pinpoint what Alex might need during upcoming medical treatments such as emotional reassurance and physical presence. | Tailored support | Dyadic health influence model (Huelsnitz et al., 2022); Thriving through social support (Feeney & Collins, 2015); Need support (Niemic et al., 2014); Responsiveness (Reis, 2014); Skilled support (Rafaeli & Gleason, 2009); Optimal matching theory (Cutrona & Russell, 1990); ^6^Social networks (Kahn & Antonucci, 1980) |
| 59b |  |  | the dyad | Billie is encouraged to brainstorm and write down support strategies that work best for them as a dyad to achieve their joint goal of reducing daily stress. |  |  |
| 59c | The dyad |  | one partner | The dyad is prompted to discuss and identify words of encouragement or actions Billie finds most motivating for the attempt to quit smoking. |  |  |
| 59d |  |  | the dyad | The dyad is guided to discuss what kind of support they as a dyad would appreciate most and judge as effective for their joint goal of eating more fiber-rich meals. |  |  |
|  |  |  |  |  |  |  |
| 60a  (3a) | One partner | **practices skills to provide support to** | the other partner | To optimize support of Billie’s stress management, Alex is asked to rehearse words of encouragement and comforting strategies in anticipation of Billie's anxiety about an upcoming medical procedure. | Support provision  skills | Social support provision (Dunkel-Schetter, & Skokan, 1990; Social support, stress and the buffering hypothesis, (Cohen & McKay, 1984); Developmental-contextual model (Berg & Upchurch, 2007); Dyadic coping: systemic-transactional model of dyadic coping (Bodenmann, 1997, 2016); Need support (Niemiec et al., 2014); Relationships motivation theory (Deci & Ryan, 2014, Ryan & Deci, 2000); Social support and health (Holt-Lunstad & Uchino, 2015); Thriving through social relationships (Feeney & Collins, 2015) |
| 60b |  |  | the dyad |  |  |  |
| 60c  (3c) | The dyad |  | one partner | The dyad is instructed to practice a supportive interaction in which Alex provides support to Billie for the goal of losing weight. |  |  |
| 60d |  |  | the dyad |  |  |  |
| 61a  (4a) | One partner | **practices communication skills to provide support to** | the other partner | Billie is instructed to participate in a communication workshop to learn how to actively listen and respond supportively when Alex talks about the challenges of adhering to a restrictive diet. |  |  |
| 61b |  |  | the dyad |  |  |  |
| 61c  (4c) | The dyad |  | one partner | The dyad is guided through role-playing exercises to practice how Alex can express support for Billie’s attempt to lose weight and how Billie could respond appropriately. |  |  |
| 61d |  |  | the dyad |  |  |  |
| 62a  (10a) | One partner | **strengthens confidence to provide support to** | the other partner | Alex is urged to remember past successes in supporting Billie, boosting Alex’s confidence to help Billie to quit smoking. |  |  |
| 62b |  |  | the dyad |  |  |  |
| 62c  (10c) | The dyad |  | one partner | The dyad is guided to recall times Billie successfully supported Alex through tough diet transitions, strengthening Billie’s confidence as a support person. |  |  |
| 62d |  |  | the dyad |  |  |  |
| Note. The numbers in parentheses indicate cross-references to intervention tasks, which are listed twice in the compendium: once in relation to health behaviors and once in relation to support behaviors. | | | | | | |
| 63a  (15a) | One partner | **recognizes and/or changes attitude towards providing support to** | the other partner | Billie is encouraged to think about how helpful the support provided will be for Alex to achieve the goal of being more active. | Support provision  skills | Social support provision (Dunkel-Schetter, & Skokan, 1990; Social support, stress and the buffering hypothesis, (Cohen & McKay, 1984); Developmental-contextual model (Berg & Upchurch, 2007); Dyadic coping: systemic-transactional model of dyadic coping (Bodenmann, 1997, 2016); Need support (Niemiec et al., 2014); Relationships motivation theory (Deci & Ryan, 2014, Ryan & Deci, 2000); Social support and health (Holt-Lunstad & Uchino, 2015); Thriving through social relationships (Feeney & Collins, 2015) |
| 63b |  |  | the dyad |  |  |  |
| 63c  (15c) | The dyad |  | one partner | The dyad is asked to focus on the positive aspects of Alex providing support for Billie’s diabetes-related lifestyle change. |  |  |
| 63d |  |  | the dyad |  |  |  |
| 64a  (25a) | One partner | **sets goals to provide support to** | the other partner | Alex is prompted to set goals for actively helping Billie to lose weight, for instance by assisting in healthy lunch preparation. |  |  |
| 64b |  |  | the dyad |  |  |  |
| 64c  (25c) | The dyad |  | one partner | The dyad is directed to set goals for Alex supporting Billie’s adherence to an exercise routine. |  |  |
| 64d |  |  | the dyad |  |  |  |
| 65a  (26a) | One partner | **commits to provide support to** | the other partner | Billie is encouraged to make a promise to support Alex in managing the side effects of chemotherapy. |  |  |
| 65b |  |  | the dyad |  |  |  |
| 65c  (26a) | The dyad |  | one partner | The dyad is directed to commit to Alex’s supporting and prioritizing Billie’s dietary needs. |  |  |
| 65d |  |  | the dyad |  |  |  |
| Note. The numbers in parentheses indicate cross-references to intervention tasks, which are listed twice in the compendium: once in relation to health behaviors and once in relation to support behaviors. | | | | | | |
| 66a  (28a) | One partner | **makes a contract for support behavior for** | the other partner | Alex is advised to set up a contract for providing support to Billie regarding Billie’s medication intake. | Support provision  skills | Social support provision (Dunkel-Schetter, & Skokan, 1990; Social support, stress and the buffering hypothesis, (Cohen & McKay, 1984); Developmental-contextual model (Berg & Upchurch, 2007); Dyadic coping: systemic-transactional model of dyadic coping (Bodenmann, 1997, 2016); Need support (Niemiec et al., 2014); Relationships motivation theory (Deci & Ryan, 2014, Ryan & Deci, 2000); Social support and health (Holt-Lunstad & Uchino, 2015); Thriving through social relationships (Feeney & Collins, 2015) |
| 66b |  |  | the dyad |  |  |  |
| 66c  (28a) | The dyad |  | one partner | The dyad is guided to make a written agreement on who takes care of the children at what times to help Alex make time for swimming practice. |  |  |
| 66d |  |  | the dyad |  |  |  |
| 67a  (29a) | One partner | **sets cues for providing support to** | the other partner | Billie is instructed to set cues such as setting an alarm on the phone to remember to assist Alex’s blood glucose measurement following meals. |  |  |
| 67b |  |  | the dyad |  |  |  |
| 67c  (29c) | The dyad |  | one partner |  |  |  |
| 67d |  |  | the dyad |  |  |  |
| 68a | One partner | **identifies own emotions while providing support to** | the other partner | Billie is instructed to reflect on own emotions while supporting Alex’s adherence to cancer treatment. |  |  |
| 68b |  |  | the dyad |  |  |  |
| 68c | The dyad |  | one partner |  |  |  |
| 68d |  |  | the dyad |  |  |  |
| Note. The numbers in parentheses indicate cross-references to intervention tasks, which are listed twice in the compendium: once in relation to health behaviors and once in relation to support behaviors. | | | | | | |
| 69a  (31a) | One partner | **plans to provide**  **support to** | the other partner | Alex is encouraged to make specific plans on when and how to support Billie’s adherence to a dietary regime; for instance, by planning to chop fresh vegetables as snacks for the coming week on Sunday mornings. | Support provision  skills | Social support provision (Dunkel-Schetter, & Skokan, 1990; Social support, stress and the buffering hypothesis, (Cohen & McKay, 1984); Developmental-contextual model (Berg & Upchurch, 2007); Dyadic coping: systemic-transactional model of dyadic coping (Bodenmann, 1997, 2016); Need support (Niemiec et al., 2014); Relationships motivation theory (Deci & Ryan, 2014, Ryan & Deci, 2000); Social support and health (Holt-Lunstad & Uchino, 2015); Thriving through social relationships (Feeney & Collins, 2015) |
| 69b |  |  | the dyad |  |  |  |
| 69c  (31c) | The dyad |  | one partner | The dyad is guided to jointly plan when and how they as a dyad support Alex’s goal to go on a run every morning. |  |  |
| 69d |  |  | the dyad |  |  |  |
| 70a  (36a) | One partner | **identifies barriers with identification of solutions (problem solvining) for providing support to** | the other partner | Being prompted to identify barriers for supporting Alex’s diabetes-specific meal planning, Billie recognizes that Billie’s own demanding work commitments may challenge continuous supportive action. |  |  |
| 70b |  |  | the dyad |  |  |  |
| 70c  (36c) | The dyad |  | one partner | The dyad is invited to identify together what prevents Billie from offering encouragement when Alex feels unmotivated to go running. |  |  |
| 70d |  |  | the dyad |  |  |  |
| 71a  (40a) | One partner | **monitors support provision of** | the other partner | After each gym session, Alex is instructed to reflect on and remember support received from Billie that made the trip to the gym easier. |  |  |
| 71b |  |  | the dyad |  |  |  |
| 71c  (40c) | The dyad |  | one partner | The dyad is encouraged to discuss Alex’s efforts in supporting Billie’s goal to increase daily water intake. |  |  |
| 71d |  |  | the dyad |  |  |  |
| Note. The numbers in parentheses indicate cross-references to intervention tasks, which are listed twice in the compendium: once in relation to health behaviors and once in relation to support behaviors | | | | | | |
| 72a  (41a) | One partner | **reviews existing social support for** | the other partner | Billie is prompted reflect on and evaluate own actions to support Alex’s diabetes management. | Support provision  skills | Social support provision (Dunkel-Schetter, & Skokan, 1990; Social support, stress and the buffering hypothesis, (Cohen & McKay, 1984); Developmental-contextual model (Berg & Upchurch, 2007); Dyadic coping: systemic-transactional model of dyadic coping (Bodenmann, 1997, 2016); Need support (Niemiec et al., 2014); Relationships motivation theory (Deci & Ryan, 2014, Ryan & Deci, 2000); Social support and health (Holt-Lunstad & Uchino, 2015); Thriving through social relationships (Feeney & Collins, 2015) |
| 72b |  |  | the dyad |  |  |  |
| 72c  (41c) | The dyad |  | one partner | Together, the dyad is invited to review the support measures Billie has already implemented during Alex's cardiac rehabilitation. |  |  |
| 72d |  |  | the dyad |  |  |  |
| 73a | One partner | **gives feedback on support provision of** | the other partner | Alex is asked to provide feedback to Billie about the helpfulness of support provided during Alex’s efforts to become more physically active. |  |  |
| 73b |  |  | the dyad |  |  |  |
| 73c | The dyad |  | one partner |  |  |  |
| 73d |  |  | the dyad |  |  |  |

Note. The numbers in parentheses indicate cross-references to intervention tasks, which are listed twice in the compendium: once in relation to health behaviors and once in relation to support behaviors.

^1^ Intervention mapping (i.e., IM): shifting perspective, ^2^ Behavioral change taxonomy (BCT): pros and cons, ^3^ BCT: Information about health consequences, IM: consciousness raising, personalized risk, ^4^ BCT: social reward (outcome or behavior), material reward, non-specific reward IM: provide contingency rewards, ^5^ BCT: remove reward, ^6^ BCT: incentive, ^7^ BCT: goal setting (behavioral outcome), IM: goal setting, ^8^ BCT: commitment, ^9^ BCT: review goals, ^10^ BCT: behavioral contract, ^11^ BCT: reduce prompts/cues, restructuring the social environment, restructuring the physical environment, IM: stimulus control, ^12^BCT: action planning, IM: implementation intentions, ^13^ BCT: problem solving: barrier identification without solution is not sufficient, IM: planning coping responses, participatory problem solving, ^14^ BCT: monitoring of behavior by others with/without feedback, IM: self-monitoring of behavior, ^15^ BCT: behavioral substitution, IM: counterconditioning, ^16^ IM: modeling, ^17^ BCT: behavioral practice/rehearsal, IM: counter conditioning, ^18^ IM: persuasive communication, coercion, ^19^ IM: persuasive communication, BCT: verbal persuasion about capabilities, ^20^ IM: punishment, BCT: punishment, ^21^ BCT: social support (practical), ^22^ BCT: social support (emotional), ^23^ BCT: social support (unspecified), ^24^ IM: mobilizing social support, mobilizing social networks

^†^Labels and definitions originate from the theoretical domains framework (i.e., TDF; Atkins et al., 2017; Cane et al., 2012)
*We refer to coach as a person who teaches techniques and strategies to assist and guide the other partner in pursuing and improving a specific health behavior. In this role, the coach is not just a teacher or trainer but also a partner who supports, challenges, and accompanies the other in achieving his/her/their specific health behavior change.
^a^ This DBCT is also known as dyadic planning (Burkert et al., 2011), ^b^ this DBCT is also known as collaborative planning (Prestwich et al., 2005)

**References**

Ajzen, I. (1991). The theory of planned behavior. *Organizational Behavior and Human Decision Processes, 50*(2), 179–211. <https://doi.org/10.1016/0749-5978(91)90020-T>

Atkins, L., Francis, J., Islam, R., O’Connor, D., Patey, A., Ivers, N., Foy, R., Duncan, E. M., Colquhoun, H., Grimshaw, J. M., Lawton, R., & Michie, S. (2017). A guide to using the theoretical domains framework of behaviour change to investigate implementation problems. *Implementation Science, 12*(1), 1-18. <https://doi.org/10.1186/s13012-017-0605-9>

Bandura, A. (2001). Social cognitive theory: an agentic perspective. *Annual Review of Psychology, 52*(1), 1–26. <https://doi.org/10.1146/annurev.psych.52.1.1>

Batson, C. D., Chang, J., Orr, R., & Rowland, J. (2002). Empathy, attitudes, and action: Can feeling for a member of a stigmatized group motivate one to help the group? *Personality and Social Psychology Bulletin, 28*(12), 1656–1666. <https://doi.org/10.1177/014616702237647>

Berkman, L. F., Glass, T., Brissette, I., & Seeman, T. E. (2000). From social integration to health: Durkheim in the new millennium. *Social Science & Medicine, 51*(6), 843–857. <https://doi.org/10.1016/S0277-9536(00)00065-4>

Berg, C. A., & Upchurch, R. (2007). A developmental-contextual model of couples coping with chronic illness across the adult life span. *Psychological Bulletin, 133*(6), 920. https://doi.org/10.1037/0033-2909.133.6.920

Berli, C., Stadler, G., Inauen, J., & Scholz, U. (2016). Action control in dyads: a randomized controlled trial to promote physical activity in everyday life. *Social Science & Medicine, 163,* 89-97. <https://doi.org/10.1016/j.socscimed.2016.07.003>

Bodenmann, G. (1997). Dyadic coping: a systemic-transactional view of stress and coping among couples. Theory and empirical findings. *European Review of Applied Psychology, 47*(2), 137–141.

Bodenmann, G. (2005). Dyadic coping and its significance for marital functioning. In T. A. Revenson, K. Kayser, & G. Bodenmann (Eds.), *Couples coping with stress: emerging perspectives on dyadic coping* (pp. 33–49). American Psychological Association. <https://doi.org/10.1037/11031-002>

Bodenmann, G., Randall, A. K., & Falconier, M. K. (2016). Coping in couples: the systemic transactional model (STM). In G. Bodenmann, A. K. Randall, M. K. Falconier (Eds.), *Couples coping with stress: a cross-cultural perspective* (pp. 5-22). Routledge. <https://doi.org/10.4324/9781315644394>

Burkert, S., Scholz, U., Gralla, O., Roigas, J., & Knoll, N. (2011). Dyadic planning of health-behavior change after prostatectomy: a randomized-controlled planning intervention. *Social Science & Medicine, 73*(5), 783–792. <https://doi.org/10.1016/j.socscimed.2011.06.016>

Cane, J., O’Connor, D., & Michie, S. (2012). Validation of the theoretical domains framework for use in behaviour change and implementation research. *Implementation Science, 7*(1), 1-17. <https://doi.org/10.1186/1748-5908-7-37>

Carvalho, T., Alvarez, M. J., Barz, M., & Schwarzer, R. (2015). Preparatory behavior for condom use among heterosexual young men: a longitudinal mediation model. *Health Education & Behavior, 42*(1), 92-99. <https://doi.org/10.1177/1090198114537066>

Cialdini, R. B., & Trost, M. R. (1998). Social influence: Social norms, conformity and compliance. In D. T. Gilbert, S. T. Fiske, & G. Lindzey (Eds.), *The Handbook of Social Psychology* (pp. 151–192). McGraw-Hill.

Cohen, S., & McKay, G. (1984). Social support, stress and the buffering hypothesis: a theoretical analysis. In A. Baum, S. E. Billie, & J. E. Singer (Eds.), *Handbook of Psychology and Health* (Vol. 4, pp. 253-267).

Cutrona, C. E., & Russell, D. W. (1990). Type of social support and specific stress: toward a theory of optimal matching. In B. R. Sarason, I. G. Sarason, & G. R. Pierce (Eds.), *Social support: An interactional view* (pp. 319-366). Wiley.

Dunkel-Schetter, C., & Skokan, L. A. (1990). Determinants of social support provision in personal relationships. *Journal of Social and Personal Relationships, 7*(4), 437-450. <https://doi.org/10.1177/0265407590074002>

Epstein, N., & Baucom, D. H. (2002). *Enhanced cognitive-behavioral therapy for couples: A contextual approach*. American Psychological Association. https://doi.org/10.1037/10481-000

Feeney, B. C., & Collins, N. L. (2015). A new look at social support: a theoretical perspective on thriving through relationships. *Personality and Social Psychology Review, 19*(2), 113–147. <https://doi.org/10.1177/1088868314544222>

Fekadu, Z., & Kraft, P. (2002). Expanding the theory of planned behaviour: the role of social norms and group identification. *Journal of Health Psychology, 7*(1), 33–43. <https://doi.org/10.1177/1359105302007001650>

Fitzsimons, G. M., Finkel, E. J., & vanDellen, M. R. (2015). Transactive goal dynamics. *Psychological Review, 122*(4), 648–673. <https://doi.org/10.1037/a0039654>

Goldsmith, D. (1992). Managing conflicting goals in supportive interaction: an integrative theoretical framework. *Communication Research, 19*(2), 264-286. <https://doi.org/10.1177/009365092019002007>

Haslam, C., Jetten, J., Cruwys, T., Dingle, G., & Haslam, A. (2018). *The new psychology of health: unlocking the social cure*. Routledge.

Holt-Lunstad, J., & Uchino, B. N. (2015). Social support and health. In K. Glanz, B. K. Rimer, & K. V. Viswanath (Eds.), *Health behavior: Theory, research, and practice*. Jossey-Bass/Wiley.

Huelsnitz, C. O., Jones, R. E., Simpson, J. A., Joyal-Desmarais, K., Standen, E. C., Auster-Gussman, L. A., & Rothman, A. J. (2022). The dyadic health influence model. *Personality and Social Psychology Review, 26*(1), 3–34. <https://doi.org/10.1177/10888683211054897>

Hughes, M., & Gove, W. R. (1981). Living alone, social integration, and mental health. *American Journal of Sociology, 87*(1), 48–74. <https://doi.org/10.1086/227419>

Kahn, R. L., & Antonucci, T. C. (1980). Convoys over the life course: attachment, roles, and social support. In P. B. Baltes, & O. Brim (Eds.), *Life-span Development and Behavior* (Vol. 3). Academic Press.

Lewis, M. A., McBride, C. M., Pollak, K. I., Puleo, E., Butterfield, R. M., & Emmons, K. M. (2006). Understanding health behavior change among couples: an interdependence and communal coping approach. *Social Science & Medicine, 62*(6), 1369–1380. <https://doi.org/10.1016>

Lewis, M. A., & Rook, K. S. (1999). Social control in personal relationships: impact on health behaviors and psychological distress. *Health Psychology, 18*(1), 63–71. <https://doi.org/10.1037/0278-6133.18.1.63>

Lyons, K. S., & Lee, C. S. (2018). The theory of dyadic illness management. *Journal of Family Nursing, 24*(1), 8-28. <https://doi.org/10.1177/1074840717745669>

Lyons, R. F., Mickelson, K. D., Sullivan, M. J. L., & Coyne, J. C. (1998). Coping as a communal process. *Journal of Social and Personal Relationships, 15*(5), 579–605. <https://doi.org/10.1177/0265407598155001>

Maddux, J. E., & Rogers, R. W. (1983). Protection motivation and self-efficacy: a revised theory of fear appeals and attitude change. *Journal of Experimental Social Psychology, 19*(5), 469–479. <https://doi.org/10.1016/0022-1031(83)90023-9>

McAlister, A. L., Perry, C. L., & Parcel, G. S. (2008). How individuals, environments, and health behaviors interact: Social Cognitive Theory. In K. Glanz, B. K. Rimer, & K. Viswanath (Eds.), *Health behavior and health education: Theory, research, and practice* (pp. 169–188). Jossey-Bass.

Miller, W. R., & Rollnick, S. (2012). *Motivational interviewing: Helping people change* (3rd ed.). Guilford Press.

Niemiec, C. P., Soenens, B., & Vansteenkiste, M. (2014). Is relatedness enough? On the importance of need support in different types of social experiences. In N. Weinstein (Ed.), *Human motivation and interpersonal relationships: Theory, research, and applications* (pp. 77-96). Springer.

O’Farrell, T. J., & Fals-Stewart, W. (2006). *Behavioral couples therapy for alcoholism and drug abuse*. Guilford Press.

O’Farrell, T. J., & Schein, A. Z. (2011). Behavioral couples therapy for alcoholism and drug abuse. *Journal of Family Psychotherapy, 22*(3), 193–215. <https://doi.org/10.1080/08975353.2011.602615>

Prestwich, A., Conner, M., Lawton, R., Bailey, W., Litman, J., & Molyneaux, V. (2005). Individual and collaborative implementation intentions and the promotion of breast self-examination. *Psychology & Health, 20*(6), 743–760. <https://doi.org/10.1080/14768320500183335>

Rafaeli, E., & Gleason, M. E. J. (2009). Skilled support within intimate relationships. *Journal of Family Theory & Review, 1*(1), 20–37. <https://doi.org/10.1111/j.1756-2589.2009.00003.x>

Reis, H. T. (2014). Responsiveness: affective interdependence in close relationships. In M. Mikulincer & P. R. Shaver (Eds.), *Mechanisms of social connection: From brain to group* (pp. 255–271). American Psychological Association. <https://doi.org/10.1037/14250-015>

Revenson, T. A. (1994). Social support and marital coping with chronic illness. *Annals of Behavioral Medicine, 16*(2), 122-130. <https://doi.org/10.1093/abm/16.2.122>

Rook, K. S. (1987). Social support versus companionship: Effects on life stress, loneliness, and evaluations by others. *Journal of Personality and Social Psychology, 52*(6), 1132–1147. <https://doi.org/10.1037/0022-3514.52.6.1132>

Rook, K. S. (2015). Social networks in later life: weighing positive and negative effects on health and well-being. *Current Directions in Psychological Science, 24*(1), 45–51. <https://doi.org/10.1177/0963721414551364>

Rosenberg, M. B. (2015). *Nonviolent communication: A language of life: Life-changing tools for healthy relationships* (3rd ed.). Puddle Dancer Press.

Rusbult, C. E., & Van Lange, P. A. (2003). Interdependence, interaction, and relationships. *Annual Review of Psychology, 54*(1), 351–375. <https://doi.org/10.1146/annurev.psych.54.101601.145059>

Ryan, R. M., & Deci, E. L. (2000). Self-determination theory and the facilitation of intrinsic motivation, social development, and well-being. *American Psychologist, 55*(1), 68–78. <https://doi.org/10.1037/0003-066X.55.1.68>

Scholz, U., & Berli, C. (2014). A dyadic action control trial in overweight and obese couples (DYACTIC). *BMC Public Health, 14*(1), 1321. <https://doi.org/10.1186/1471-2458-14-1321>

Sterba, K. R., de Vellis, R. F., Lewis, M. A., Baucom, D. H., Jordan, J. M., & de Vellis, B. (2007). Developing and testing a measure of dyadic efficacy for married women with rheumatoid arthritis and their spouses. *Arthritis Care & Research, 57*(2), 294-302. <https://doi.org/10.1002/art.22538>

Su, Y.-L., & Reeve, J. (2011). A meta-analysis of the effectiveness of intervention programs designed to support autonomy. *Educational Psychology Review, 23*(1), 159–188. <https://doi.org/10.1007/s10648-010-9142-7>
