# Supplementary material for: Compendium of dyadic behavior change techniques v2.0: results from a Delphi study
Source: Ann Behav Med. 2025 Nov 7;59(1):kaaf080. doi: 10.1093/abm/kaaf080 (PMC12597882; doi:10.1093/abm/kaaf080)
Supplement: kaaf080_Supplementary_Data [file kaaf080_supplementary_data.zip › Suppl.zip!/ESM2 Distal mechanisms of action.docx]

ESM 2. Collection of distal mechanisms of action of DBCTs proposed by experts during the Delphi Process

(bold = mentioned at least twice)

Social exchange processes

- **Social support (informational and emotional)**
- Support Quality
- Supportive behaviors
- Social influence
- Social/relational identity
- Social comparison
- Social contagion
- Social norms
- Cultural influences or norms

Individual and Dyadic Regulation

- **Self-efficacy due to modeling**
- **Motivation**
- Autonomous or controlled motivation (SDT)
- Competence
- **Autonomy/ Couple autonomy**
- Goal salience
- Goal commitment
- Attitude
- Enthusiasm
- Coping strategies
- Empowerment
- Stress reduction
- Shared appraisals
- Shared goals
- Shared norms
- Shared mental representation of the goals
- Collaborative problem-solving, cooperative conflict resolution style
- Sense of responsibility (by the agent) to try other behavior change techniques

Interpersonal / Relationship processes

- **Accountability**
- mutual respect
- emotion co-regulation, emotion regulation
- **Commitment**
- **Intimacy/closeness**
- **Relationship satisfaction**/closeness/cohesion or similar factors related to emotional ties in the dyad
- Feelings of connectedness to the partner and relationship
- Relatedness
- Sense of the dyad as a unit whose outcomes rise and fall together
- **Reciprocity**
- Mutuality
- **Empathy**
- **Perspective-taking**
- Shared values
- Responsiveness
- (dyad) Psychological safety
- Changes/developments in partners' attachment styles
- Trust
